# Neuronal Parasitism, Early Myenteric Neurons Depopulation and Continuous Axonal Networking Damage as Underlying Mechanisms of the Experimental Intestinal Chagas' Disease

**DOI:** 10.3389/fcimb.2020.583899

**Published:** 2020-10-15

**Authors:** Mayra Fernanda Ricci, Samantha Ribeiro Béla, Michele Macedo Moraes, Maria Terezinha Bahia, Ana Lia Mazzeti, Anny Carolline Silva Oliveira, Luciana Oliveira Andrade, Rafael Radí, Lucía Piacenza, Rosa Maria Esteves Arantes

**Affiliations:** ^1^Departament of Pathology, Federal University of Minas Gerais, Belo Horizonte, Brazil; ^2^Departament of Biological and Exact Sciences, Federal University of Ouro Preto, Ouro Preto, Brazil; ^3^Department of Morphology, Federal University of Minas Gerais, Belo Horizonte, Brazil; ^4^Departament of Bioquímica, Facultad de Medicina, Center for Free Radical and Biomedical Research, Universidad de La Republica Montevideo, Montevideo, Uruguay

**Keywords:** *Trypanosoma cruzi*, pathogenesis, intestinal chagas's disease, experimental model, nitric oxide (NO), reactive species, nitrergic neurons, enteric nervous system (ENS)

## Abstract

There is a growing consensus that the balance between the persistence of infection and the host immune response is crucial for chronification of Chagas heart disease. Extrapolation for chagasic megacolon is hampered because research in humans and animal models that reproduce intestinal pathology is lacking. The parasite-host relationship and its consequence to the disease are not well-known. Our model describes the temporal changes in the mice intestine wall throughout the infection, parasitism, and the development of megacolon. It also presents the consequence of the infection of primary myenteric neurons in culture with *Trypanosoma cruzi* (*T. cruzi*). Oxidative neuronal damage, involving reactive nitrogen species induced by parasite infection and cytokine production, results in the denervation of the myenteric ganglia in the acute phase. The long-term inflammation induced by the parasite's DNA causes intramuscular axonal damage, smooth muscle hypertrophy, and inconsistent innervation, affecting contractility. Acute phase neuronal loss may be irreversible. However, the dynamics of the damages revealed herein indicate that neuroprotection interventions in acute and chronic phases may help to eradicate the parasite and control the inflammatory-induced increase of the intestinal wall thickness and axonal loss. Our model is a powerful approach to integrate the acute and chronic events triggered by *T. cruzi*, leading to megacolon.

## Introduction

Chagas' disease (CD) is a zoonotic long-term human infectious disease caused by the protozoan *Trypanosoma cruzi*, which is naturally transmitted to animals and people by triatomine insects found only in the Americas. This neglected disease is transmitted through blood transfusion, organ transplantation, consumption of parasite-contaminated food, and vertically from mother to child. It affects eight million people worldwide, mostly in Latin America, leads to high morbidity, and to a potentially life-threatening pathology of the heart and gastrointestinal tract (WHO, 2018).

The disease is divided into two phases. The acute phase is defined by high parasitemia, which usually lasts up to 3 months and involves mild non-specific symptoms. The next phase, the chronic undetermined phase, in which infected individuals are initially asymptomatic lasts for decades. After this period, patients may develop clinical symptoms of the infection, characterized by the cardiac (30–50%), digestive (4.5–15%), or cardio-digestive (2–10%) forms of CD (Rassi et al., 2012; Pérez-Molina and Molina, 2018).

The mechanisms by which some patients develop anatomic-pathological alterations of megacolon remain unclear (De Oliveira et al., 1998; Teixeira et al., 2011), and much less investigated than chronic Chagas' heart disease (Brener et al., 2000; Tarleton, 2001; Bonney and Engman, 2008; Burgos et al., 2010; Teixeira et al., 2011; Machado et al., 2012; Dutra et al., 2014). Several descriptive studies of human megacolon samples have been published (Jabari et al., 2011, 2012, 2014). However, these approaches did not allow the proposition of a pathogenic mechanism linking the acute to the chronic phase of the disease throughout the natural history of digestive Chagas' disease, which is one of the objectives of the current work.

In humans, the megacolon alterations include hypertrophy of the circular muscular layer, focal inflammatory reactions in the vicinity of the myenteric plexus and the muscular layer, and fibrosis of the myenteric plexus (Koberle and de Alcantara, 1960; Koberle, 1968). The intestinal symptoms include dysphagia, regurgitation, severe constipation, and alterations in barium enema (De Oliveira et al., 1998).

Few studies aimed to investigate how and when the denervation of myenteric plexus happens and its consequences to the contraction and peristalsis of the gut. In the acute phase of the disease, organ damage occurs as a direct consequence of the parasite's host cell invasion, followed by the inflammatory response generated by the activation of the innate immune response, as well as by Th1 pro-inflammatory cytokines, such as IL-12, tumor necrosis factor α and interferon γ (Laranja et al., 1956; Shikanai-Yasuda and Carvalho, 2012). These cytokines activate the production of inducible nitric oxide synthase (iNOS) and nitric oxide (NO) by macrophages, which may contribute to the neurotoxic effects observed (Gazzinelli et al., 1992; Aliberti et al., 1999; Garcia et al., 1999; Pinto et al., 2002; Arantes et al., 2004).

Knowledge about the chronic digestive disease is less clear. The acute death of enteric neurons (ganglionic denervation), both *in vivo* and *in vitro* (Arantes et al., 2004; Almeida-Leite et al., 2007), results from the interaction of the parasite and neuronal host cells that contribute to the development of the chronic digestive disease. Although neuronal cell death was reported in human megavisceras previously (Koberle and de Alcantara, 1960; González Cappa et al., 1987; DeFaria et al., 1988; Adad et al., 2001, 2013; da Silveira et al., 2005; Nascimento et al., 2010), studies of the mechanisms of neuronal death *in vivo* and *in vitro* are scarce (Tanowitz et al., 1992; Almeida-Leite et al., 2007).

Production of reactive oxygen species (ROS) and other oxidative stress-related pathways have been described in cardiomyocytes (Dias et al., 2017; Estrada et al., 2018) and may have a role in cardiac CD (Gupta et al., 2009; Lopez et al., 2018). It remains unknown if these mechanisms operate in enteric neurons and how the neuronal anti-oxidant mechanism contributes to the susceptibility, or the selective vulnerability of enteric neurons to oxidative stress-induced damage (Bubenheimer et al., 2016).

Importantly, discrimination between the experimental acute and chronic phases of the disease have not been appropriately investigated (Guillén-Pernía et al., 2001; Cruz et al., 2016), since most studies did not focus on disease progression and avoid specific age correlation between mice and humans (Dutta and Sengupta, 2016). This is even more important when considering human CD's chronic manifestations as they depend on the long-term infection (decades), usually occurring at patients' midlife or later, which would correspond to around 15 months of age for mice (Flurkey et al., 2007). Several studies investigated up to 3 months post-infection and, therefore, their conclusions are only applicable to the acute or subacute phases of the human disease, when the anatomical pathological findings are still not present (Guillén-Pernía et al., 2001; Moreira et al., 2011; Gupta and Garg, 2013; Massocatto et al., 2017).

Factors such as the extremely long evolution in human patients, the gut length, the anatomic and functional complexity of the intestinal wall, and the scarce opportunities for accessing representative megavisceras samples have hampered research investigating the pathogenesis of the intestinal disease. Therefore, the current work explores the causes of megacolon in an experimental model previously described (Campos et al., 2016), while reproducing aspects of the human megacolon. These research questions are extremely important, since very little is known about this form of Chagas disease. Indeed, the intramural intestinal nervous system modifications and the consequences to the dynamics of neuromuscular plasticity, triggered by the inflammatory response elicited by *T. cruzi* infection, still need to be systematically studied in this long-term experimental model.

To elucidate the mechanisms underlying the intestinal structural progressive changes that lead to the megavisceras, we evaluated diverse intestinal histological and molecular aspects during the evolution of the infection in mice at several time intervals between the acute (11 days post-infection–d.p.i.) and chronic phases (3, 7, 12, and 15 months post-infection–m.p.i.). We also infected primary cultures of enteric neurons and smooth muscle cells with *T. cruzi* to evaluate its effects on neuronal damage.

Since oxidative stress and mitochondrial damage have been implicated in neurodegenerative diseases (Cenini et al., 2020), we investigated their role in myenteric neuronal damage. The translational approach and the description of the timeline of the anatomic pathological substrate brings light to the pathogenesis of this neglected disease and may help future development of tissue-damaging markers, effective eradicating drugs, as well as preventive and early interventions in infected and vulnerable individuals.

## Materials and Methods

This study was carried out in strict accordance with the recommendations of the Guide for the Care and Use of Laboratory Animals of the Brazilian National Council of Animal Experimentation (http://www.cobea.org.br/) and Federal Law 11.794 (October 8, 2008). The animal study was reviewed and approved by The Institutional Committee for Animal Ethics of Federal University of Minas Gerais (CEUA/UFMG–Licenses 262/2016 and 25/2018).

### Experimental Chagasic Megacolon Induced in Mice by Chronic *T. cruzi* Infection

#### Mice

We used 4-week old Swiss female mice, supplied by the Bioterium of the Institute of Biological Sciences of UFMG, and kept in the Bioterium of the Federal University of Ouro Preto (UFOP) in plastic cages, in a room with controlled dry temperature (24°C) under a light / dark cycle of 14/10 h, and access to water and to conventional mouse food (Nuvilab® Nuvital, Brazil).

#### Experimental Design

Our model induced the chronic phase in mice infected with Y *T. cruzi* strain by administering a single oral dose of benznidazole (500 mg/kg) at 11 d.p.i. This treatment scheme is unable to cure Y strain infected mice, but prevents the acute death of around 30% of animals and guarantees that the circulating parasites can reach the intestinal wall and trigger the local pathology (Campos et al., 2016).

The infected animals were randomly divided into two subgroups: (1) infected mice euthanized on the 11th day post-infection (d.p.i.), which we called infected acute phase (IAP) group. Euthanasia occurred 2 days after the peak of parasitemia, according to the model previously described by our laboratory (Campos et al., 2016); (2) mice which infected on the same day as the previous group and treated orally with a single dose of 500 mg/kg body weight of benznidazole (Lafepe, Brazil). These mice were maintained up to 15 months post-infection (m.p.i.) and composed the infected chronic phase (ICP) group. Animals were euthanized at 3, 7, 12, and 15 m.p.i. (ICP3, ICP7, ICP12, and ICP15 groups, respectively) so that the analysis of several parameters of megacolon development could be performed.

The uninfected age-matched control groups were maintained in the same conditions and euthanized as: control of acute phase (CAP) or acute phase (AP); and control of chronic phase (CCP) or chronic phase (CP) at the appropriate months indicated as CCP3, CCP7, CCP12, CCP15 and ICP3, ICP7, ICP12, ICP15.

**Figure 1:**
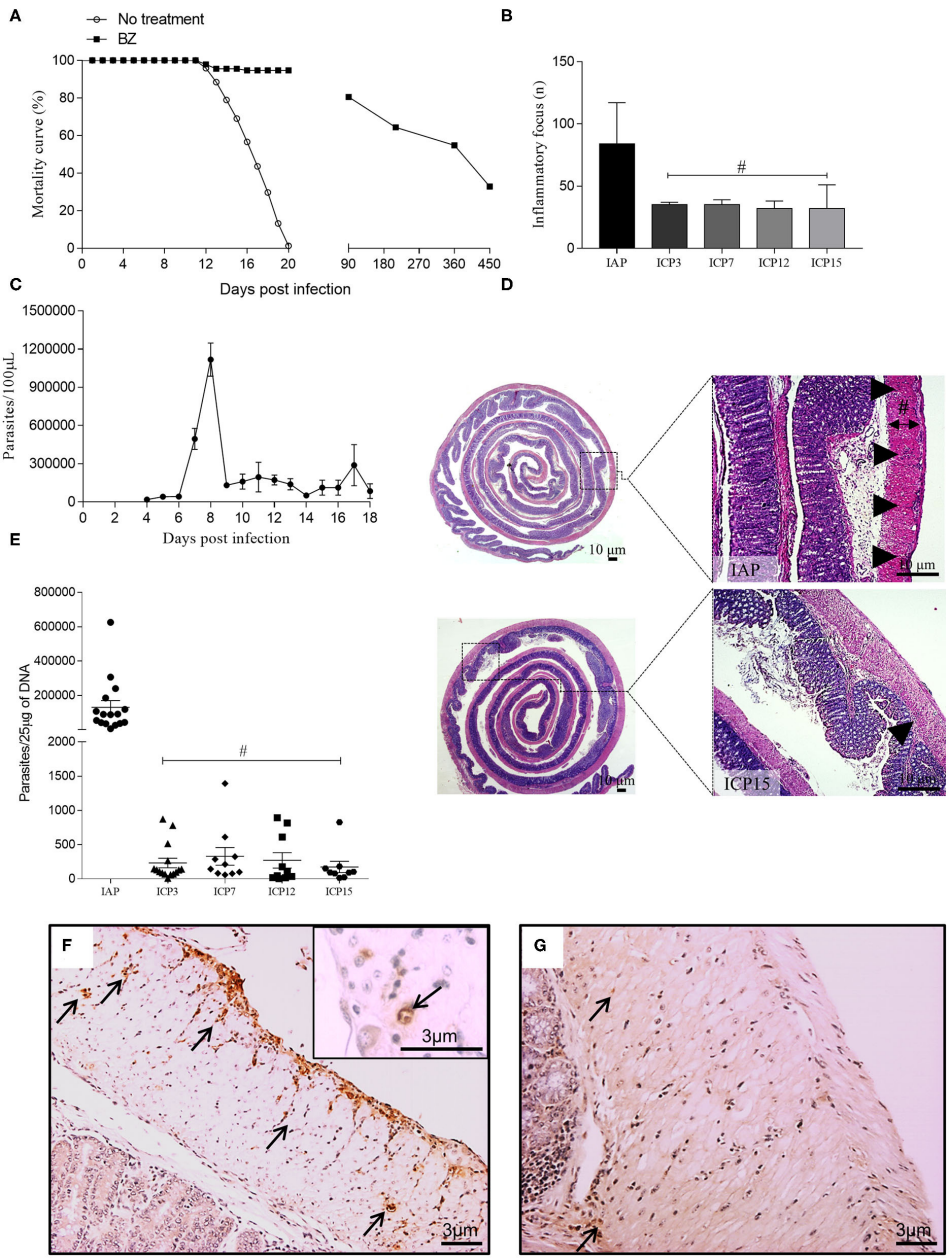
Mortality curve, infection parameters, and characterization of the inflammation associated with parasitism throughout the infection. Swiss female mice were infected with 50,000 *T. cruzi* strain Y trypomastigotes. **(A)** Survival curve: The mice in the control group and those treated with benznidazole (BZ) were followed throughout the time of infection. Control animals (no treatment) showed 100% mortality up to 20 d.p.i.; **(B)** Inflammatory foci: following HandE staining, 30 fields presenting inflammatory foci in the colon of infected mice were analyzed using an Olympus BX51 optical microscope with a 20X objective. Inflammatory foci were identified by the number of cells (at least 10 cells) and counted for the IAP, ICP3, ICP7, ICP12, and ICP15 groups. (*n* = 3 mice). Data are representative of two independent experiments. Statistical analysis: ANOVA one-way with Tukey's *post-hoc* tests, after logarithmic transformation. Difference in relation to the infected acute phase group (IAP), *P* ≤ 0.05 (#). Data are shown as mean and standard deviation (SD); **(C)** The peak of parasitemia was reached at 8 d.p.i.; **(D)** Sagittal section of the intestine showing the IAP (upper panel) and chronic infected (ICP15.; lower panel) stages of infection. The distribution of inflammation in the acute phase presents as diffuse, coalescent foci whereas in the chronic phase, it persists as smaller foci. The thickness of the inner muscular layer was measured from the submucosal layer to the inner edge of the outer muscular layer (black line; Scale bar: 10 μm. 1X and 5X objective; the Sharp symbol indicates the width of the inner muscular layer); **(E)** DNA of the parasite: a sample of ~0.5 cm was taken from the proximal and distal colon of acute (IAP) and chronic (ICP3, ICP7, ICP12, ICP15) mice. Tissues were macerated and DNA extraction was performed, followed by PCR to amplify the parasite's DNA. (*n* = 9–16 mice). Data are representative of two independent experiments. Statistical analysis: one-way ANOVA with Student-Newman-Keuls *pos-hoc* test. Time difference from acute (IAP), *P* < 0.001 (#). Data are shown as mean and SD; **(F,G)** Immunolabelling of paraffin-embebbed tissues from infected colons using anti-*T. cruzi*; **(F)** Acute phase, presence of amastigote nests along the colon (arrows); **(G)** Chronic phase, at 15 m.p.i., rare foci and less intense labeling (arrows). Scale bar = 3 μm. 10X and 20X objective. The arrowheads indicate the inflammatory infiltrate and the arrows indicate immunostaining for anti-*T. cruzi* at 11d.p.i. (acute phase) and 15 m.p.i. (chronic phase). Infected Acute Phase (IAP), Infected Chronic Phase 3 (ICP3), Infected Chronic Phase 7 (ICP7), Infected Chronic Phase 12 (ICP12), Infected Chronic Phase 15 (ICP15).

In a parallel experiment, 10 infected and benznidazole-untreated animals were checked daily for parasites in their blood until the parasitemia ceased completely (see Figure 1A), thus allowing us to determine the exact time of treatment (11 d.p.i.). All animals infected and benznidazole-treated were examined daily and checked for blood parasites at 3, 7, 12, and 15 m.p.i. The benznidazole-treated animals did not show signs of sickness, pain distress, suffering or moribund conditions.

#### Experimental Procedures

Inoculum preparation for animal infection was performed according to previously described methodology (Brener, 1962). Infection of the animals was done intraperitoneally with 50,000 blood trypomastigotes of *T. cruzi* Y strain, at the Laboratory of Parasitic Diseases, School of Medicine of the UFOP, Brazil. Infection of all inoculated animals was confirmed by the presence of parasites in fresh blood 4 days after inoculation.

Two independent experiments were performed involving a total of 201 female Swiss mice were used in the *in vivo* study. One hundred and fifty-one mice were inoculated intraperitoneally with 50,000 blood trypomastigotes of the *T. cruzi* Y strain. Ten animals were destined to the construction of the parasitemia curve (Figure 1A); 10 were euthanized at 11 d.p.i (IAP group); and 131 were treated with benznidazole (at the 11th day) and euthanized during the chronic phase at 3, 7, 12, and 15 m.p.i. Fifty animals composed the uninfected control group, 10 composed the AP group and 40 constituted the CCP groups at the times described in the previous Experimental Design section.

#### Parasitemia and Survival Curve

Parasitemia was determined by the method described previously (Ducci and Pizzi, 1949) and modified according to Brener (1962). Five microliters of blood were collected from the tip of the tail and parasite count was performed under the optical microscope (400X) in 50 random fields using a 22 × 22 mm coverslip. Mortality rate was evaluated daily in order to build a survival curve.

#### Histopathological Analysis

The colon of the mice was collected, separated from the mesentery, and washed in PBS (0.01 M phosphate-buffered saline, pH 7.2) to remove fecal content. For histology, the entire colonic segment was extended with the serosa in contact with the filter paper to avoid mucosal damage. The segments were transferred to Bouin's solution plus 2% glacial acetic acid for 5 min. The pre-fixed colon was rolled up like a swiss roll with the mucosal side facing inwards to form rolls from the distal portion (anus) to the proximal end (cecum), as described before (Arantes and Nogueira, 1997). The rolls were kept in 10% formaldehyde for 1 week and then sectioned in half to trim the edges so that, after routine paraffin embedding, the microtomy resulted in samples of the entire length of the colon for subsequent H&E staining, Masson, and immunohistochemistry. Fragments of 0.5 to 1 cm were taken from the proximal and distal colon to be frozen and processed for the extraction of parasite DNA. The animal hearts were collected and fixed in 10% formaldehyde in order to be examined later.

#### Inflammatory Infiltrate Quantification

Using a bright field microscope with a 20X objective, we counted the absolute number of inflammatory foci of 30 fields from each colon (H&E stained). Inflammatory foci were characterized by the presence of at least 10 inflammatory cells in the intestinal muscle layer. A sample of three representative animals from all infected groups was evaluated at all time intervals (11 d.pi. and 3, 7, 12, and 15 m.p.i.).

#### Immunohistochemistry

Consecutive histological sections measuring 4 μm in thickness were obtained from the colon rolls of infected and uninfected animals. The sections were deparaffinized and rehydrated for the subsequent blockade of endogenous peroxidase with 30% methanol and 5% hydrogen peroxide. To block non-specific reactions, we used 2% Bovine Albumin (BSA, Inlab, Brazil) with 0.1% Triton X-100 (Sigma-Aldrich, USA) in PBS, followed by three PBS washes. To block non-specific binding of secondary antibodies, the sections were incubated with 1:20 goat normal serum (SNC, Cripton Biotechnology, Brazil) in PBS and subsequently incubated, in a humid chamber at 4°C overnight, with the following primary antibodies: anti-PGP9.5 (rabbit, 1: 500) (Cedarlane, USA); anti-α-actin (rabbit, 1: 500) (Epitomics, USA); or anti-*T. cruzi* (rabbit, 1: 5,000) (Provided by Prof. Maria Terezinha Bahia, UFOP). After PBS washes, the secondary antibody complex (goat anti-rabbit and goat anti-mouse) was added, and then incubated with the pre-diluted streptavidin peroxidase complex (KIT DAKO, LSAB, K0675, USA) for 30 minutes each in a humid chamber at 37°C. The reaction was developed by 3, 3'-diaminobenzidine tetrahydrochloride (DAB, D5637, Sigma-Aldrich, USA) and the sections were counterstained in diluted Harris Hematoxylin solution. Next, the slides were dehydrated at increasing alcohol concentrations, diaphanized in xylol, and permanently mounted with Entellan (Merck, USA).

#### Obtaining Images

For photographic documentation and to obtain the images needed for morphometric analysis, the slides were photographed using the Olympus BX51 direct light optical microscope equipped with Image-Pro Express 4.0 software (Media Cybernetics, USA) with a resolution of 1,392 × 1,040 pixels. Images were transferred via a Cool SNAP-Proof Color camcorder to a computer-attached video system using Image-Pro Express version 4.0 for Windows (Media Cybernetics, USA).

#### Morphometric Analysis

Images (20X objective) were acquired from PGP 9.5 immunolabelled samples to analyze the innervation profiles of the myenteric plexus ganglionic neurons and of the intramuscular (internal muscle). In each field, the ganglia were manually delimited to obtain the total ganglion area and the area immunolabelled with PGP 9.5 within the ganglion was automatically measured (μm^2^). Myenteric neuronal damage was determined as PGP9.5 immunolabelled l area/myenteric ganglia area. Data were expressed as mean and standard deviation.

The area of the nerve profiles immunolabelled with PGP 9.5 was automatically measured (μm^2^) and the total area of the internal muscle layer was delimited and measured manually in each field (μm^2^). Intramuscular innervation density was determined as PGP 9.5 immunolabelled intramuscular innervation/muscular inner layer area. Data was expressed as mean and standard deviation.

Images (40X objective) were acquired exclusively from the inner circular muscle layer in PGP 9.5 immunostained samples. The area (μm^2^) of the inner smooth muscle was delimited and measured as already described. The total number of cell nuclei was counted in the same fields using the Image J 1.52 program (NIH, USA) as parameter of the number of smooth muscle cells. The ratio muscle inner layer area/nuclei number indirectly indicates the average area of the intestinal smooth muscle cell and its variation throughout the study was determined. Images (10X objective) were obtained by sampling the full length of the colon from the control and infected mice at all time intervals studied (11 d.pi., 3, 7, 12, and 15 m.p.i.). For each image, three measurements (μm) of the inner muscle layer thickness were obtained using the Image J 1.52 software (NIH, USA). The inner muscle layer was defined by the boundaries of the submucosal layer and the outer muscle layer (Figure 1D). We expressed the data as mean and standard deviation of the thickness of the inner muscular layer as the index of colon wall thickness. The ratio PGP 9.5 positive intramuscular innervation area/ thickness of the inner muscular layer was determined.

Morphometrical analyses were performed by KS300 at the Department of Pathology, ICB / UFMG. All the analyzes were conducted in blinded experiments.

#### Quantitation of *T. cruzi* in Colon Samples (Real-Time PCR for Parasite DNA)

Tissue parasitism was evaluated in frozen samples submitted to the detection of parasite DNA by real-time polymerase chain reaction (PCR). Colon samples were thawed, minced and digested with proteinase K (20 mg/mL, Invitrogen™, USA) and DNA extraction by Promega Wizard® Genomic DNA Purification kit following the manufacturers' instructions. PCR reaction were performed in 10 μL containing 50 ng of genomic DNA (2μL), 5 μL of SYBR® Green PCR Mastermix (Applied Biosystems) and either 0.35 μM for *T. cruzi* DNA-specific primers or 0.35 μM of murine-specific tumor necrosis factor α (TNFα) primers and water. The primers for *T. cruzi* DNA (TCZ-F 5′ -GCTCTTGCCCACAMGGGTGC-3′, where M =A or C and TCZ R 5′ -CCAAGCAGCGGATAGTTCAGG-3′) amplify a 182-bp. Primers for murine TNFα (TNF-5241 5′-TCCCTCTCATCAGTTCTATGGCCCA-3′ and TNF5411 5′-CAGCAAGCATCTATGCACTTAGACCCC-3′) amplify a 170-bp product (Cummings and Tarleton, 2003).

Cycles of amplification were carried out in a 7500 Fast Real-Time PCR System (Applied Biosystems). The cycling program consisted of an initial denaturation at 95°C for 10 min, followed by 40 cycles of 94°C for 15 s and 62°C for 1 min with fluorescence acquisition. Amplification was immediately followed by a melt program with an initial denaturation of 15 s at 95°C, cooling to 60°C for 1 min and then a stepwise temperature increases of 0.3°C/s from 60 to 95°C. Each 96-well reaction plate contained standard curve and two negative controls. Standard curves were produced from a 10-fold DNA dilution of epimastigotes of *T. cruzi* Y strain and DNA from colon tissue of non-infected mouse, ranging from 1 × 10^6^ to 1 parasites and equivalent/25 μg of tissue DNA. Negative controls consisted of a reaction with *T. cruzi*-specific or murine-specific primers without DNA and also with non-infected mouse tissue DNA. Each DNA sample was quantified in duplicate. The efficiencies of amplification were determined automatically by the 7500 Fast Real-Time PCR Software. qPCR showed amplification efficiencies >90% and a regression coefficient (*r*^2^) of 0.98.

### Primary Cultures of Mouse Myenteric Neurons

Thirty-six mice C57BL 6 from 6 to 8 weeks were euthanized to remove the full length of the large intestine. The segment was processed, as explained below, and the cells isolated from the colon muscle wall were used for *in vitro* assays. Briefly, after removal, the large intestine segment was placed in Krebs solution (126 mM NaCl, 5 mM KCl, 1.25 mM MgSO4, 1.25 mM KH2PO4, 2.5 mM CaCl, 25 mM NaHCO3, 11 mM Glucose) as described before (Wahba et al., 2016). The intestinal fragment was sectioned along the mesenteric border to obtain a flat segment of tissue, whose edges were carefully distended and fixed with entomological pins to facilitate delamination of the layers (mucosa, submucosa, and serosa) and expose the muscular layer where the Auerbach's myenteric plexuses are laid (Smith et al., 2013). The tissue was incubated for 15 min at 37°C in a 1 mg/ml solution of collagenase type II-S (Merck, USA) under shaking and manually homogenized by inversion every 5 min. The fragments were centrifuged at 200 × g for 5 min at 4°C and washed with Hanks/HEPES buffer solution for 5 min under stirring, followed by centrifugation at 200 × g for 5 min at 4°C. Trypsin solution (0.25%; Merck, USA) was added at 37°C for 10 min, followed by stirring for 15 min and manual homogenization by inversion every 5 min.

Next, the preparation was further suspended using a glass Pasteur pipette and the suspension centrifuged 200 G for 5 min at 4°C. The supernatant was removed and was added to 1 ml of sterile culture medium (Minimum Essential Medium; 31095-029; Gibco, Invitrogen, USA) enriched with 10% fetal bovine serum (FBS;16140-071; Gibco, Invitrogen, USA) and the following antibiotics: penicillin (100 IU/ml; P4333, Sigma-Aldrich, USA) and streptomycin (10,000 μg/ml; P4333, 36; Sigma-Aldrich, USA). The suspended cells were plated in a 48-well culture dish on Matrigel-coated 9mm glass coverslips (354234 BD; Franklin Lakes, USA) at a minimum density of 2x10^4^ cells per well (Arantes et al., 2000). The resulting preparations will be referred as primary myenteric neurons cultures.

#### *In vitro T. cruzi* Infection

The *T. cruzi* strains DM28c (*T. cruzi* I type) and Y (*T. cruzi* II type) were used in infection experiments (Zingales, 2018). Trypomastigote forms were cultured and purified using kidney epithelial lineage (VERO) cells, as described previously (Braga et al., 1993). After 6 days of culture, the parasites collected were centrifuged at 150 G for 10 min at room temperature and counted in a Neubauer chamber, centrifuged at 450 G for 10 min at 4°C and suspended 90% DMEM medium in 10% fetal calf serum (FCS) and added to cultures. These experiments were performed in collaboration with Prof. Dr. Luciana O. Andrade from the Morphology Department, ICB/UFMG and Prof. Dr. Lucia Piacenza from the Department of Biochemistry, Montevideo Medical School (UDELAR). Trypomastigotes of both strains were added to the primary culture of neurons at a ratio of 10:1 and the cultures were incubated at 37°C with 5% CO_2_ and studied at 24, 48, and 72 h post-infection (h.p.i.).

#### Evaluation of Oxidant Production by Infected Cells

A general measure of NO formation was performed using the probe DAF-FM (4-Amino-5-Methylamino-2′, 7′-Difluorofluorescein Diacetate) (Invitrogen, USA), which fluoresces green upon oxidation. For this, primary cultures of mouse enteric neurons grown on coverslips in 48-well plates, as described above, were infected or not with *T. cruzi* strains Y or DM28c trypomastigotes and followed up for 48 and 72 h.p.i. Then, the coverslips were washed once with PBS and exposed to DAF-FM (2 μM in PBS) for 15 min at 37°C. After the infection, we also used Ionomycin (1 μM) (Sigma-Aldrich, USA), which induces Ca^2+^ influx and interferes with nNOS activity through calmodulin binding, and evaluated its effect in the DAF-FM fluorescence. IFNγ (0.1 mg/mL) (Sigma-Aldrich, USA) was added to control cultures for 48 h before submitted to DAF-FM assays. After incubation, cells were imaged using a fluorescence microscope (Nikon Eclipse TE 200) equipped with a digital camera. Green fluorescence was captured using a filter cube with an excitation wavelength of 540 nm (band pass, 25 nm) and an emission filter of 605 nm (band pass, 55 nm).

A general measure of oxidant formation was performed using the probe CM-H_2_DCFDA [5- (and-6) -chloromethyl-2′, 7′-dichlorodihydrofluorescein diacetate, acetyl ester] (Molecular Probes, USA), which fluoresces upon oxidation (Gupta et al., 2009). For this, primary cultures of mouse enteric neurons grown on coverslips in 48-well plates were infected or not with *T. cruzi* strains Y or DM28c trypomastigotes and followed up for 48 and 72 h.p.i. Then, the coverslips were washed once with PBS and exposed to CM-H_2_DCFDA at a final concentration of 1 μM in PBS. Immediately after the addition of CM-H_2_DCFDA, the 48-well plate was read using the fluorimeter Synergy^TM^ 2 (Biotek®, USA) at 37°C to monitor the probe's oxidization rate, with excitation and emission wavelengths of 485 and 520 nm, respectively. Data were analyzed using the program Gen5^TM^. Probe oxidation curves were used to calculate the slope and are expressed as Relative Fluorescence Units (RFU)/minute.

#### Mitochondrial Membrane Potential in *T. cruzi*-Infected Primary Enteric Neurons, Glia, and Smooth Muscle

Mitochondrial membrane potential (Δψ) was measured using the J-aggregate-forming lipophilic cation JC-1. For this, primary cultures of mouse enteric neurons grown on coverslips in 48-well plates were infected or not with *T. cruzi* strains Y or DM28c trypomastigotes and followed up for 72 h.p.i. Then, the coverslips were washed once with PBS and exposed to JC-1 (0.5 μM) (Molecular Probes, USA) and incubated at 37°C for 1 h before rinsed with Krebs buffer. Green fluorescence (JC-1-monomers at the cytosol) and red fluorescence (mitochondrial matrix JC-1-aggregates) were detected using fluorescence microscopy as described above, at a fixed exposure time.

#### Immunofluorescence and Photographic Documentation

Primary cultures of mouse enteric neurons submitted to the above-described conditions and grown on coverslips were fixed with 4% buffered paraformaldehyde at room temperature for at least 1 h. To identify neuronal bodies and neurites and facilitate their quantification we used anti-PGP 9.5 (Rabbit, 1:500; Cedarlane, USA), anti-β-tubulin III (Mouse, 1:800; Millipore, USA) and anti-nNOS (Rabbit, 1:500; Epitomics, USA). Smooth muscle cells were identified using the anti-α actin antibody (Rabbit, 1: 400; Abcam, USA). For the immune-labeling of other structures we used the following antibodies: anti-3-NO_2_-tyrosine (Rabbit, 1:200; provided by Dr. Rafael Radi), an antibody that specifically detects the protein bound 3-Nitrotyrosine (NO_2_Tyr) (Brito et al., 1999); anti-MnSOD2 (Mouse, 1:200; Santa Cruz, USA) and anti-*T. cruzi* (Rabbit, 1: 5,000; provided by Prof. Maria T. Bahia, UFOP). Identification of cell nuclei *in vitro* was performed by the fluorescence-emitting probe Hoechst H33342 (Invitrogen, USA), which binds to nuclear DNA and allows the optimal visualization of parasite and host nuclear morphology. The coverslips were analyzed under the Olympus BX51 fluorescence microscope and images were obtained using Image-Pro Express 4.0 software (Media Cybernetics, USA).

#### Fluorescence Intensity Measurements

Using viable, *in vitro* intact cells with well-stained nuclei, we quantified the fluorescence intensities of neuronal bodies and neuritic network (24 h.p.i., 40X objective, anti β-tubulin III) as well as α-actin filaments (24, 48, 72 h.p.i.; 20X objective, anti-α-actin) in at least eight images randomly photographed of control and infected groups, obtained from at least two coverslips with primary cultures of mouse enteric neurons, from two independent experiments. DAF-FM and JC-1 (20X objective), anti-3-NO_2_-tyrosine (40X objective) fluorescence intensity was analyzed in at least five images selected by the presence of neuronal bodies, of control and infected groups, obtained from two coverslips, in duplicate experiments.

JC-1 fluorescence of enteric neurons grown on coverslips was expressed as an indicator of potential-dependent accumulation in mitochondria, indicated by a fluorescence emission shift from green (~525 nm) to red (~590 nm) detected upon excitation and filtering using an Olympus BX51 fluorescence microscope equipped with a dual-bandpass. We evaluated the images obtained to find the proportion of red fluorescence to green fluorescence. The micrographs were analyzed for fluorescence intensity in arbitrary units using Image J 1.52 (Bereiter-Hahn, 1975).

#### Expression of MnSOD2 by Western Blot

The expression levels of mitochondrial MnSOD2 were evaluated by western blot. Briefly, control and infected cultures were treated with trypsin from porcine pancreas (Sigma-Aldrich, USA) and lysed in buffer Tris-EDTA (10 and 1 mM, respectively) containing 0.1% of Triton X-100. For immunoblotting, samples were separated by SDS–PAGE (15%), transferred onto the nitrocellulose membrane and blocked with non-fat dry milk (3% w/v) in PBS (pH 7.4) for 1 h. The membranes were incubated with a monoclonal anti-MnSOD2 antibody (1: 200) (Santa Cruz, USA). Primary and secondary antibodies were diluted 1: 1000 in Tween-20 (0.1% v/v) in PBS (pH 7.4) and incubated overnight at 4°C. The membranes were then washed in Tween-20 (0.1%, v / v) in PBS and incubated for 1 h with anti-mouse IgG antibody (1:15.000) (IR Dye-800 conjugated; LI-COR Biosciences). After washing, immunoreactive proteins were visualized with an infrared fluorescence detection system (Odyssey, LI-COR Biosciences).

### Statistical Analysis

The Shapiro-Wilk test revealed that the parameters evaluated did not show a significant departure from normal distribution, except for parasite DNA (Figure 1E), which was submitted to a logarithmic transformation and analyzed as normally distributed data. *In vivo* experiments of infection were repeated twice in which at least five mice were analyzed per group and time point. *In vitro* experiments were repeated twice and at least two coverslips of each were used for the analysis.

Comparisons between means were made using unpaired student's tests (i.e., Control vs. Infected). One-way analyses of variance (ANOVAs) were used to compare multiple treatments *in vitro* experiments. C (CTRL), CTRL + IFN (IFNγ), DM28c, DM28c + INF and CTRL, CTRL + INF, Y, Y + INF or at the time points (11 d.p.i.and 3, 7, 12, and 15 m.p.i.). A two-way analysis of variance (ANOVA) was used to compare treatments (Control vs. infected) along the ime points (11 d.p.i and 3, 7, 12, and 15 m.p.i.). When a significant *F*-value was found, we performed a *post-hoc* test according to the coefficient of variation (CV): Tukey (CV ≤ 15%) or Student-Newman-Keuls (CV> 15%). The α level was set at 0.05. Data are shown as mean ± standard deviation (SD).

## Results

### Parasite-Induced Acute Inflammation, Persistence of *T. cruzi* DNA, and Chronic Inflammation of the Intestinal Wall Are Correlated to Megacolon Development

Experimental infection with the *T. cruzi* Y strain led to a high acute mortality rate (100%) of infected animals, which was initiated immediately after the parasitemia peak at 8–9 d.p.i until 18 d.p.i. (Figures 1A,C). Our model was designed to prevent the acute death of 30% of the infected mice by administering a single intra-peritoneal sub-therapeutic dose of benznidazole to guarantee that the circulating parasites could reach the intestinal wall (Figures 1B,D) and trigger local pathology. The surviving animals that followed through the chronic phase were monitored up to 15 m.p.i. and euthanized at 3, 7, 12, and 15 m.p.i. to study the sequential installation of the structural changes associated with megacolon development. Most benznidazole-treated animals died between the first and the third month of infection (~50%) and the rest of them died during the experiment until 450 d.p.i. (Figure 1A). Once the acute phase was over (absence of circulating blood parasites), all surviving animals remained asymptomatic. The surviving treated-animals remained asymptomatic throughout the chronic phase of the infection and did not show signs of sickness, pain distress, suffering, or of moribund conditions. At this phase, the *T. cruzi* infection has been confirmed by positive results of qPCR assay to detect *T. cruzi* DNA.

All animals euthanized by 11 d.p.i. (acute phase) presented high parasitemia at 8 d.p.i. (1.2 × 10^6^ trypomastigotes/mL; Figure 1C) and unequivocal tissue parasitism in the colonic segment (Figures 1E,F), which was associated with intense and focal inflammatory infiltrates dispersed throughout the extension of the colon (Figures 1B,D, arrowheads). We quantified the inflammatory foci from the distal (center of the intestine roll) to the proximal border of the colon at 11 d.p.i. up to 15 m.p.i. Inflammation was distributed randomly in the intestinal wall, in foci detected in the serosa, the muscularis propria (inner and outer layers), and the myenteric ganglia (Auerbach plexus) (Figure 1D). We observed a significant decrease in the chronic phase's inflammation score in comparison with the inflammation scores obtained for the acute phase time points (Figure 1B). There was an increase in CD4+ cells in both phases compared to their respective controls (Supplementary Figures 1A–C). In contrast, compared to their respective controls, CD8+ cells significantly decreased in infected animals at the acute phase and increased at the chronic phase (*P* < 0.09). However, the number of inflammatory foci was maintained from 3 to 15 m.p.i., indicating the persistence of a chronic inflammatory process (Figure 1B).

At earlier time points (11 d.p.i. and 3 m.p.i.), there were degenerative changes of neuronal and glial cells inside the intramural ganglia as well as integral or disrupted nests of parasites, which were frequently observed inside smooth muscle and glia cells (Supplementary Figure 1E, inset). Inflammation and parasitism interrupted the normal circular and longitudinal layering of smooth muscle cells by depositing transudates and necrosis foci (Figure 1D).

Treatment with benznidazole induced a marked decrease in parasitemia, which was no longer detected in the surviving animals after 18 d.p.i. (Figure 1C). The parasite was observed in the H&E stained sections and in the immunohistochemical data from the intestinal wall collected at the acute phase (Figure 1F). However, we did not easily observe parasites in samples obtained at the chronic phase (Figure 1G). We also detected the parasite's DNA in samples collected throughout the intestine wall. Although PCR quantification revealed an abrupt decrease in parasite DNA at the chronic phase compared to the acute phase, small amounts were still detected until 15 m.p.i. (Figure 1E). According to the parasite blood circulation criteria, these animals underwent infection chronicity and presented chronic intestinal inflammation (Figure 1D, arrowheads), which were correlated with the progressive colonic structural changes observed in our model of megacolon development.

### Early Acute Ganglionic Neuronal Death Is Accompanied by a Progressive Decrease of Axonal Profiles' Density, Increased Intestinal Wall Thickness, and Smooth Muscle Cell Hypertrophy

**Figure 2:**
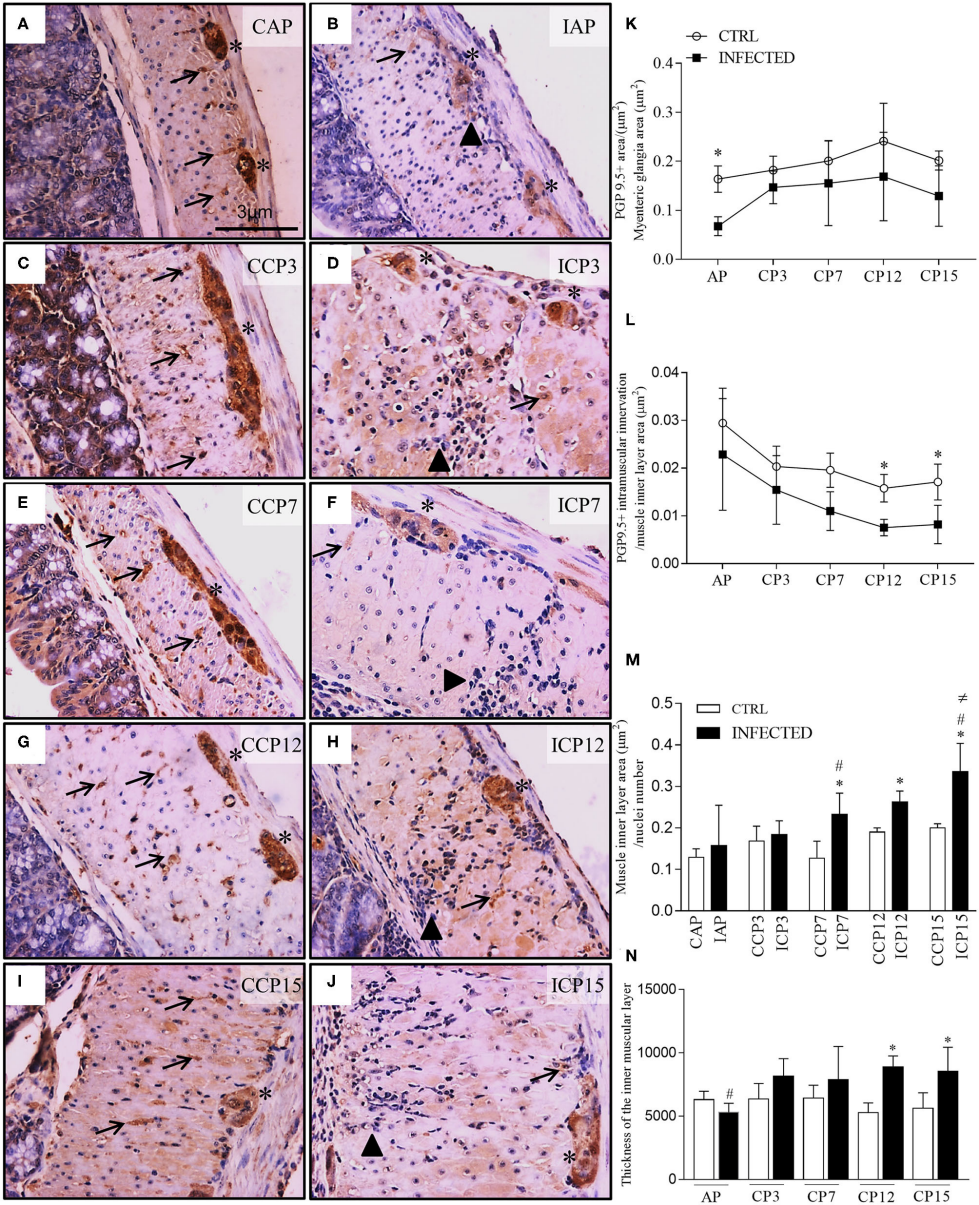
Ganglionic neuronal damage, intramuscular denervation, and phenotype changes of intestinal muscular propria during megacolon development. **(A–J)** ganglia and intestinal muscle layer of Swiss mice infected with 50,000 *T. cruzi* strain Y trypomastigotes obtained from animals at the acute (CAP, controls; IAP, infected) and chronic (CCP, controls; ICP3, ICP7, ICP12, and ICP15 as 3, 7, 12, and 15 months post-infection, respectively) phases and immunostained for PGP 9.5. Scale bar: 3 μm. 40X objective; **(K–M)** Morphometrical analysis of innervation changes. Ten images of the myenteric plexus and internal muscular layer of each animal were acquired with a 40X objective. Statistical analysis: Two- way ANOVA with Student-Newman-Keuls tests. Results are expressed as the mean and standard deviation (SD) of two independent experiments; **(K)** PGP 9.5. + area / myenteric ganglia total area (μm^2^). Difference in relation to the respective control groups, *P* ≤ 0.05 (*) (*n* = 4 mice); **(L)** PGP 9.5. + intramuscular innervation/muscle inner layer area (μm^2^). Difference in relation to the control group, *P* ≤ 0.05 (*). (*n* = 3 mice). Data are representative of two independent experiments. **(M)** Muscle inner layer area (μm^2^) / nuclei number (*n* = 3–8 mice). Difference in relation to the control group, *P* ≤ 0.05 (*). Difference in time in relation to 11 d.p.i and 3 m.p.i., *P* ≤ 0.05 (#). Difference over time in relation to all times in infected animals, *P* ≤ 0.05 (≠); **(N)** Thickness of the inner muscular layer (μm). Ten images of each animal, in HandE, were acquired with a 10X objective; three measurements of the thickness of the muscular layer were made in each image (*n* = 5 mice). Data are representative of two independent experiments. Difference in relation to each respective control group, *P* ≤ 0.05 (*). Difference in time compared to the other times in infected animals, *P* ≤ 0.05 (#). Arrows indicate immunostaining for PGP 9.5 of nerve fibers. Asterisks indicate immunostaining of neuronal bodies in the myenteric plexus ganglia. Arrowhead indicates inflammatory infiltrate. Acute Phase (AP), Chronic Phase (CP 3, 7, 12, and 15).

Pathological damage to the enteric nervous system (ENS) was studied throughout the development of the experimental megacolon by conventional H&E staining (Supplementary Figure 1). Immunohistochemistry of neuronal bodies of the myenteric ganglia and intramuscular nerve fibers (PGP 9.5-positive) as well as in the muscle inner layer (Figures 2A–J, arrows), were also evaluated.

Colonic samples, collected at 11 d.p.i. and 3 m.p.i., showed an expressive loss of neuronal PGP 9.5-positive cells when compared to paired controls. We observed degenerative changes of ganglion cells (Figures 2B,D, asterisk), associated with intense and frequent pan-mural peri-ganglionic inflammation (polymorph and mononuclear cells) and edema (Figures 2B,D, arrowheads). Transudate, congestion, and necrotic-degenerative changes of smooth muscles were also detected at these time points (Figures 2B,D). Chronic inflammation and vacuolar degenerative changes of smooth muscle cells were noticed mostly in the internal muscle layer by 7 m.p.i. up to 15 m.p.i. (Figures 2F,H,J).

We detected a decrease in neuronal PGP 9.5-positive staining in the myenteric ganglia of infected in comparison to non-infected animals (*P* < 0.001), indicating neuronal damage occurring as early as 11 d.p.i. (Figure 2K). Staining was maintained from 3 to 15 m.p.i. (*P* = 0.003) but not at 7, 12, and 15 m.p.i., when samples did not differ from those evaluated at earlier time points (11 d.p.i and 3 m.p.i.). These results suggest that structural neuronal ganglionic damages occur at the beginning of the infection and do not change with time, despite a slight variation observed at 3 m.p.i.

We also examined (Figures 2A–H) and quantified (Figure 2L) PGP 9.5-positive transverse profiles that represent the axonal intramuscular innervation. There was a marked decrease in the intramuscular PGP 9.5-immunolabeled area along time in both control and infected groups (*P* < 0.001) and between the control and infected groups (*P* < 0.002). This difference is explained by a trend observed at 7 m.p.i. (*P* = 0.052), and significance at 12 m.p.i. (*P* = 0.0013) and 15 m.p.i. (*P* = 0.049).

To further explain the evolution and the mechanism of the post-infection colon modifications, we also quantified the area of individual smooth muscle cells in the inner muscle layer of the intestinal wall (Figures 2A–J,M). The average area of smooth muscle cells was significantly increased at 7, 12, and 15 m.p.i. compared to their paired non-infected controls. The chronic phase differed significantly from the acute phase (*P* ≤ 0.05) and from the 3 m.p.i. time point (*P* ≤ 0.05). From 7 m.p.i. there was a progressive increase in the average area of smooth muscle cells in the infected animals only (Figure 2M), indicating a progressive or cumulative change of these cells.

The thickness of the colonic muscularis propria increased significantly in comparison to the acute phase at 15 m.p.i. (Campos et al., 2016). The intermediate time intervals revealed that the thickness of the smooth muscle internal layer increased significantly by 12 and 15 m.p.i. in infected animals compared to their controls. We also observed a significant and progressive increase of the smooth muscle internal layer's thickness from 3 to 15 m.p.i. in infected animals (Figure 2N; *P* ≤ 0.05).

### Deranged Smooth Muscle α-Actin Is Noticed Throughout Megacolon Development

**Figure 3:**
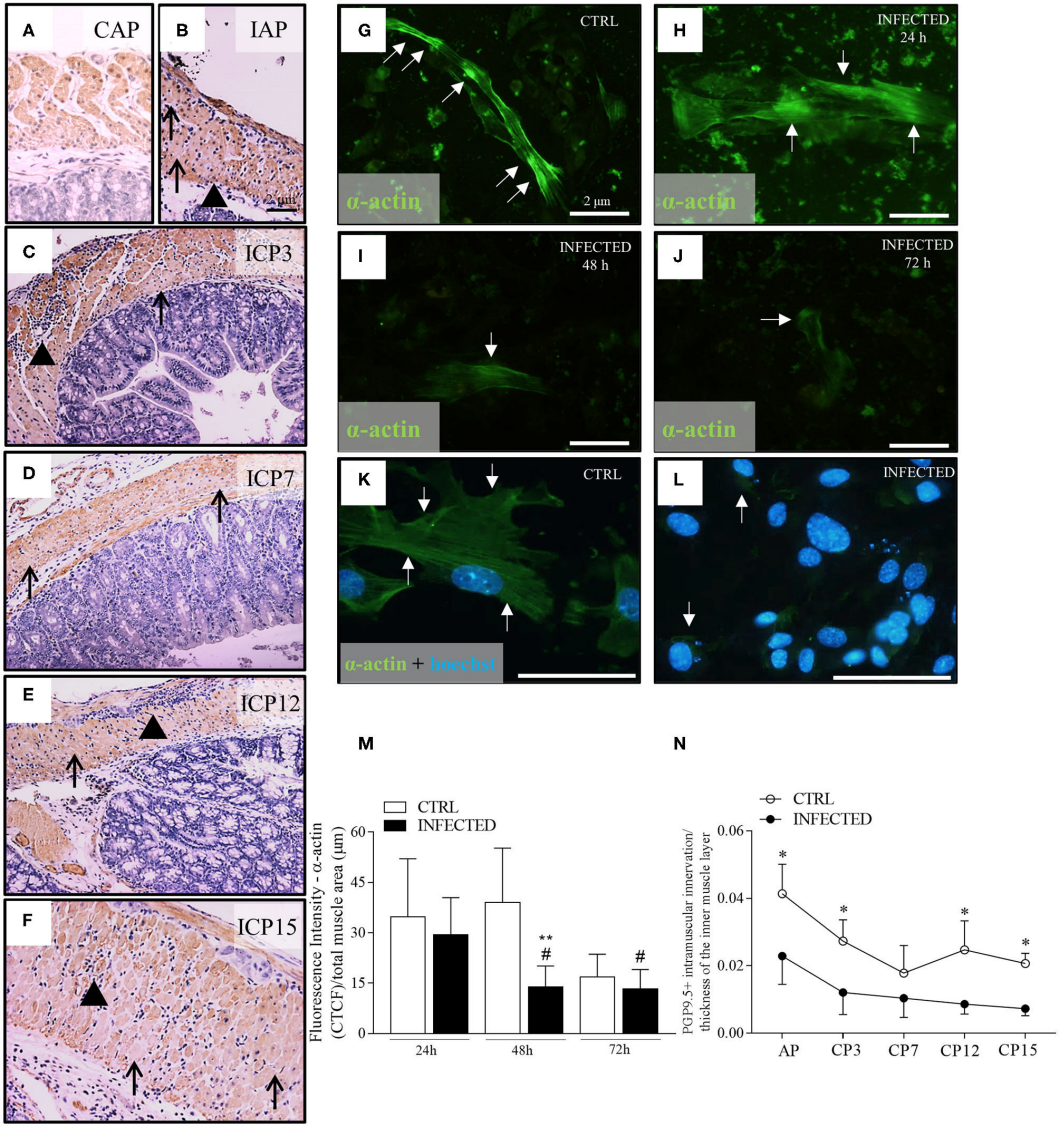
Deranged smooth muscle α-actin immuno-expression *in vivo* and *in vitro*. **(A)** Colon control samples from Swiss mice were immunostained with anti-α-actin **(B–F)** Colon samples obtained from Swiss mice infected with 50,000 *T. cruzi* Y strain trypomastigotes were immunostained with anti-α-actin. Colon samples were obtained from animals in the acute phase (IAP) and chronic phase ICP (3, 7, 12, and 15 months post-infection-m.p.i.). Scale bar: 2 μm. 10X objective. **(G–L)** Anti-α-actin immunofluorescence in control (CTRL) and infected primary enteric neuron cultures at 24, 48, and 72 h post-infection (h.p.i.). **(G–J)** Scale Bar: 2 μm. 20X objective; **(K,L)** Scale Bar: 2 μm. 40X objective. **(M)** Quantitative analysis of fluorescence intensity throughout infection (fluorescence Intensity of α-actin/total muscle area). (*n* = 3 mice). Data are representative of two independent experiments. **(N)** PGP 9.5 + intramuscular innervation/thickness of the inner muscle layer (*n* = 4). Data are representative of two independent experiments, in triplicate coverslips. Statistical analysis: ANOVA two-way with Student-Newman-Keuls tests. *Difference in relation to the control group, *P* ≤ 0.05. **Difference in relation to the respective control group, *P* < 0.01. ^#^Difference in time in relation to the 24 h.p.i., *P* < 0.05. Data are shown as mean and standard deviation (SD). Arrowheads indicate the inflammatory infiltrate. Black arrows indicate a decrease and/or absence of immunostaining for anti-α-actin in the internal muscle. White arrows indicate fluorescence for anti-α-actin. **(K,L)** Nuclear staining with Hoechst 33342.

Evaluating the damage of muscle fibers, we found that acute and chronic inflammation correlated to the irregular, weak, or absent staining of α-actin filaments in some inner layer cells of smooth muscle (Figures 3A–F, black arrows). These abnormalities occurred more frequently at later time points and may reflect cumulative effects on these cells' function. Next, we studied the effect of *T. cruzi* infection in smooth muscle cells present in primary enteric neuronal cultures up to 72 h post-infection (h.p.i.; Figures 3G–L). The typical α-actin expression pattern shown in Figure 3G changed to irregular and weak staining at 72 h.p.i. (Figure 3J). We also noticed the presence of parasite nests by Hoechst staining inside α-actin-positive cells as early as 48 h.p.i. (Figure 3L, white arrows). The intensity of α-actin immunofluorescence decreased significantly in the infected cultures along time, with marked differences at 48 h.p.i. (Figure 3M; *P* ≤ 0.05).

The *in vivo* and *in vitro* α-actin expression changes shown herein may be an additional factor leading to intestinal dysfunction, besides the increase of the thickness of the muscular *propria* and the progressive and marked loss of nerve profiles. Indeed, we observed a significant decrease in the innervation/thickness ratio between infected and control groups throughout the experiment (*P* < 0.001) (Figure 3N). This observation indicates that intramuscular innervation decreases in the hypertrophic muscle wall that, in turn, becomes composed of deranged smooth muscle cells with decreased α-actin expression.

Another structural parameter revealing the impairment of the colon's muscular wall was the progressive deposition of interstitial connective tissue (Masson's Trichrome) in the submucosa and in the muscular layer of infected animals. While samples from control animals showed thin fibers of connective tissue around intact smooth muscle cells and myenteric ganglia, the samples of infected animals exhibited disrupted periganglionic connective tissue. A disordered and augmented deposition of collagen in the interstitium of the hyperplasic smooth muscle was also observed (Supplementary Figures 1J–O, arrows).

### *In vitro* Evidence for Damages to the Enteric Nervous System and Neuronal Death

*In vivo* experiments indicated an early and non-progressive ganglionic damage with neuronal depopulation associated with the acute phase of the disease (Figure 2). In order to unveil the mechanisms of neuronal death, we prepared a primary culture of myenteric neurons in a mixed background of smooth muscle cells. These cultures were infected or not (control) with *T. cruzi* trypomastigotes and followed up for 72 h.

**Figure 4:**
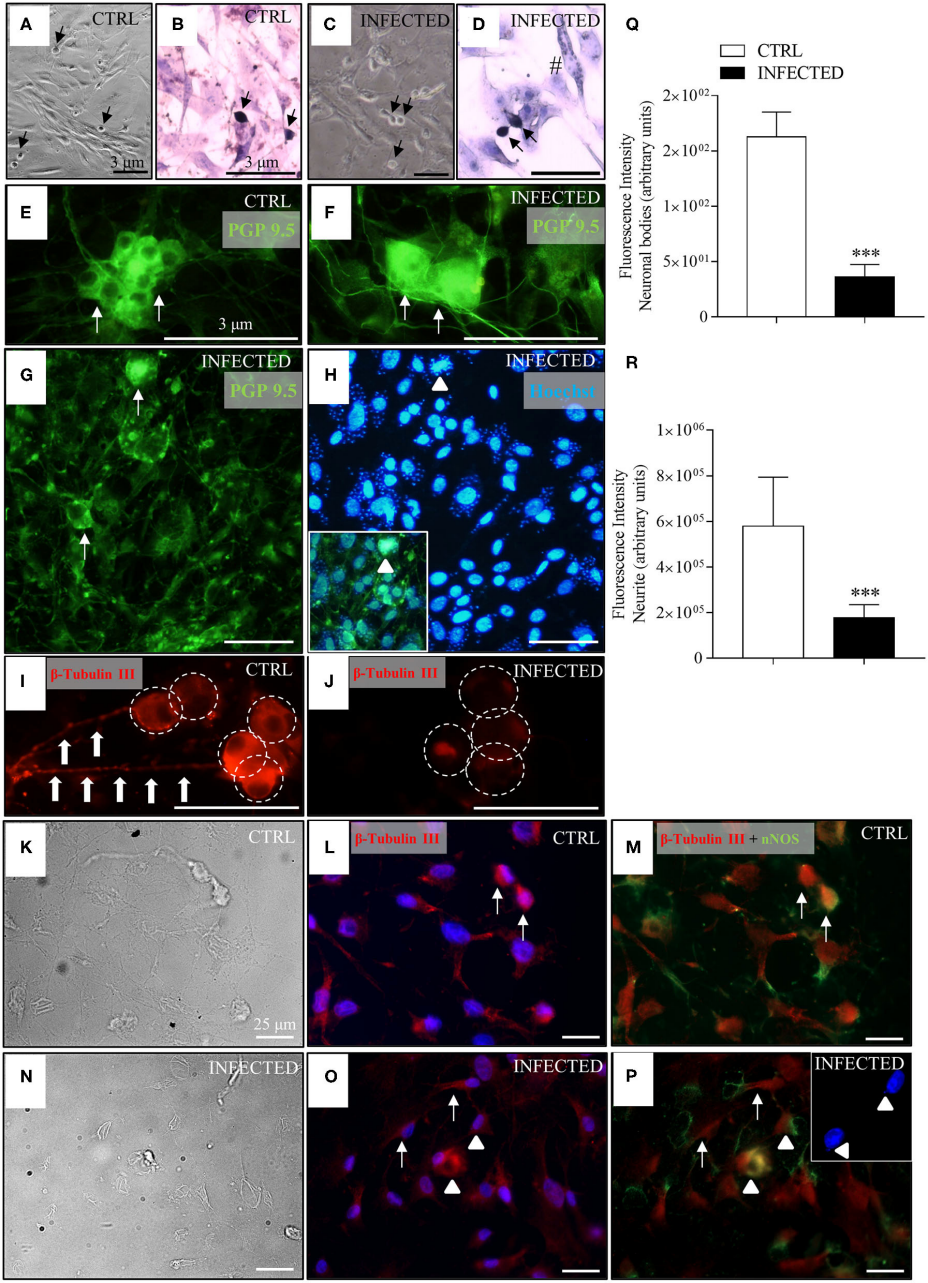
Effect of *T. cruzi* infection of enteric neuron cultures on neuronal bodies and neurite network. Neurons removed from the myenteric plexus were dissociated, plated, and infected or not with trypomastigotes of the *T. cruzi* strains Y and Dm28c. Cultures were analyzed at 24, 48, and 72 h post-infection (h.p.i.) after they were fixed using a 4% buffered paraformaldehyde solution. The coverslips were photographed in phase contrast [**(A)**, control (CTRL) and **(C)**, infected]; stained with Giemsa [**(B)**, CTRL and **(D)**, infected]; immunostained with anti-PGP 9.5 [in green; **(E)**, CTRL and **(F**–**H)**, infected] and anti-β-tubulin III [in red; **(I)**, CTRL and **(J)**, infected]; **(H)** Nuclear staining with Hoechst 33342 (in blue). Notice in the inset the presence of Hoechst fluorescent amastigotes labeled with PGP 9.5 inside neuronal cell bodies (arrowheads); β-tubulin and nNOS double-stained neurons (arrowheads) and correspondent phase-contrast images [**(K–M)**, CTRL; **(N–P)**, infected]; **(O,P)** confocal images, with arrows indicating β-tubulin stained neurons. The double nNOS and β-tubulin infected neurons are indicated by arrowheads. The inset shows Hoechst fluorescent (in blue) amastigotes inside the neurons (arrowheads). **(A,C)** 10X objective; **(B,D,G,H)** 20X objective. **(E,F,I,J)** 40X objective. Scale bar: 3 μm. **(K-P)** 40X objective. Scale bar: 25 μm **(Q)** Fluorescence intensity of anti-β-tubulin III labeled neuronal bodies; **(R)** Fluorescence intensity of anti-β-tubulin III labeled neurites (*n* = 4). Representative of two independent experiments in duplicate coverslips). Images were obtained an Olympus BX51 fluorescence microscope, 40X objective. Statistical analysis: Student *t*-test. Difference in relation to the control group, *P* < 0.001 (***). Data are shown as mean and standard deviation (SD). The arrows indicate neurons, arrowhead indicates the presence of parasites inside the neurons, large arrows indicate neuritic varicosities, and the white circles indicate neuronal bodies.

In control cultures, smooth muscle and accessory cells outnumbered neuronal cells and neurites, as identified by their morphological aspects under bright field (Figure 4A), when stained with Giemsa (Figure 4B), and upon immunolabelling with the neuronal markers PGP 9.5 (Figure 4E) and β-tubulin III (Figure 4I). Neurons were arranged in small groups or observed as isolated ovoid and birefringent cell bodies with expanding neurites firmly adhered to the smooth muscle cell monolayer, forming a dense network in control cultures (Figure 4I, large arrows).

In infected cultures, parasites were observed mostly inside smooth muscle cells and inside neuronal cells at 48 h.p.i. (Figure 4H, inset, arrowheads), some of which exhibiting accentuated degenerative changes in their cellular bodies (Figure 4G, arrows). Immunolabelling for β-tubulin III showed a significant decrease in fluorescence intensity of neuronal bodies, neurite network, and varicosities (beaded rosary aspect) at 24 h.p.i. (Figure 4J) in comparison with non-infected cultures (Figures 4I,Q,R).

Several neuronal cells were immunostained with β-tubulin III and nNOS antibodies (Figures 4K–P), indicating the presence of nitrergic neurons. The parasites' nuclei, stained by Hoechst, were mainly seen inside the cell bodies of nitrergic neurons (Figure 4P, inset), although they were also observed in a few neurons not stained for nNOS.

### Production of Reactive Nitrogen Species (RNS) and Reactive Oxygen Species (ROS) by Enteric Neurons in Response to *T. cruzi* Infection

**Figure 5:**
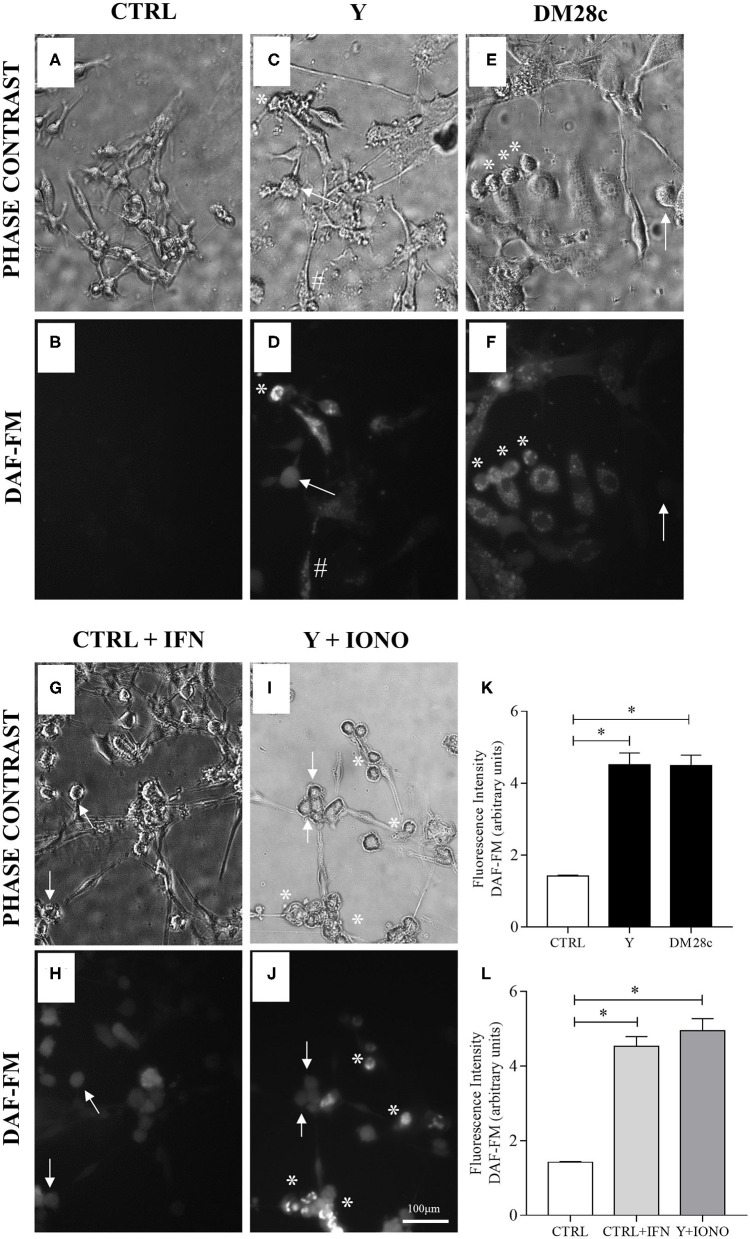
Neuronal nitric oxide production in myenteric neuronal cultures indicated by DAF-FM probe oxidation. Neurons removed from the myenteric plexus were dissociated, plated, and infected or not with *T. cruzi* strains Y and Dm28c trypomastigotes for 72 h. Then, the coverslips were washed and exposed to DAF-FM. **(A)** Control cultures (CTRL) in phase contrast and **(B)** DAF fluorescence (DAF). No oxidation of DAF-FM is observed; **(C,D)** Y strain infected cultures (Y); **(E,F)** Dm28c strain infected cultures (DM28C). Infection produced greater oxidation in cells with neuronal morphology and that were are infected (asterisk); **(G,H)** Control cultures treated with IFNγ (CTRL + IFN). Neuronal cells activated with IFNγ showed increased oxidation of DAF-FM; **(I,J)** Y strain infected and ionomycin-treated cultures (Y + IONO). Activation of cells with Ca^2+^ ionophore (IONO) increased oxidation of DAF-FM; **(K,L)** Quantification of DAF-FM fluorescence intensity. *n* = 6. Representative of three independent experiments in duplicate coverslips. Scale bar: 100 μm. 20X objective. Student *t*-test. Difference in relation to the control group, *P* < 0.05 (*). Data are shown as mean and standard deviation (SD). Asterisks indicate infected and DAF-fluorescent neurons. Arrows indicate neuronal bodies without infection. The sharp symbol indicates infected accessory cell.

To investigate the production of RNS in the primary cultures of myenteric ganglionic neurons, which contained smooth muscle cells, we infected or not these cultures with trypomastigotes of the Y and Dm28c strains of *T. cruzi*. The control cultures (Figures 5A,B) and infected cultures (Figures 5C–F) were followed up to 48 and 72 h.p.i., when parasite invasion and multiplication inside neuronal cells could be observed. NO production was assayed with DAF-FM, which reacts with NO species yielding a highly fluorescent product (Balcerczyk et al., 2005). Cultures exposed to *T. cruzi*, no matter the parasite strain, presented NO production in infected neuronal cells by 72 h.p.i., but not in non-infected neighboring cells. Additionally, invaded non-neuronal cells were discretely DAF-FM positive (Figures 5D,F, arrows, sharp symbol). Non-infected cultures were weakly DAF-FM positive but, when treated with IFN**γ**, neuronal DAF-FM fluorescence was markedly increased, suggesting these neurons are able to generate NO species (Figures 5G,H). Infected neurons of cultures treated with ionomycin, a calcium ionophore, also increased the DAF-FM fluorescence (Figures 5I,J, asterisk). The neurons of infected cultures that did not get infected with the trypomastigotes did not react (Figure 5J, arrows). DAF-FM fluorescence intensity was also quantified in neuronal cell bodies (Figures 5K,L).

Analysis of reactive oxygen species (ROS) levels produced upon infection of enteric neuronal cultures with the Y strain of *T. cruzi* was performed using the CM-H_2_-DCFDA, and the resulting fluorescence measured at 48 and 72 h.p.i. No significant difference was observed in the amount of the oxidized probe in the infected cultures compared to controls (Figure 7C).

### Infection of Primary Myenteric Neuronal Cultures With *T. cruzi* Induces Mitocondrial Depolarization

**Figure 6:**
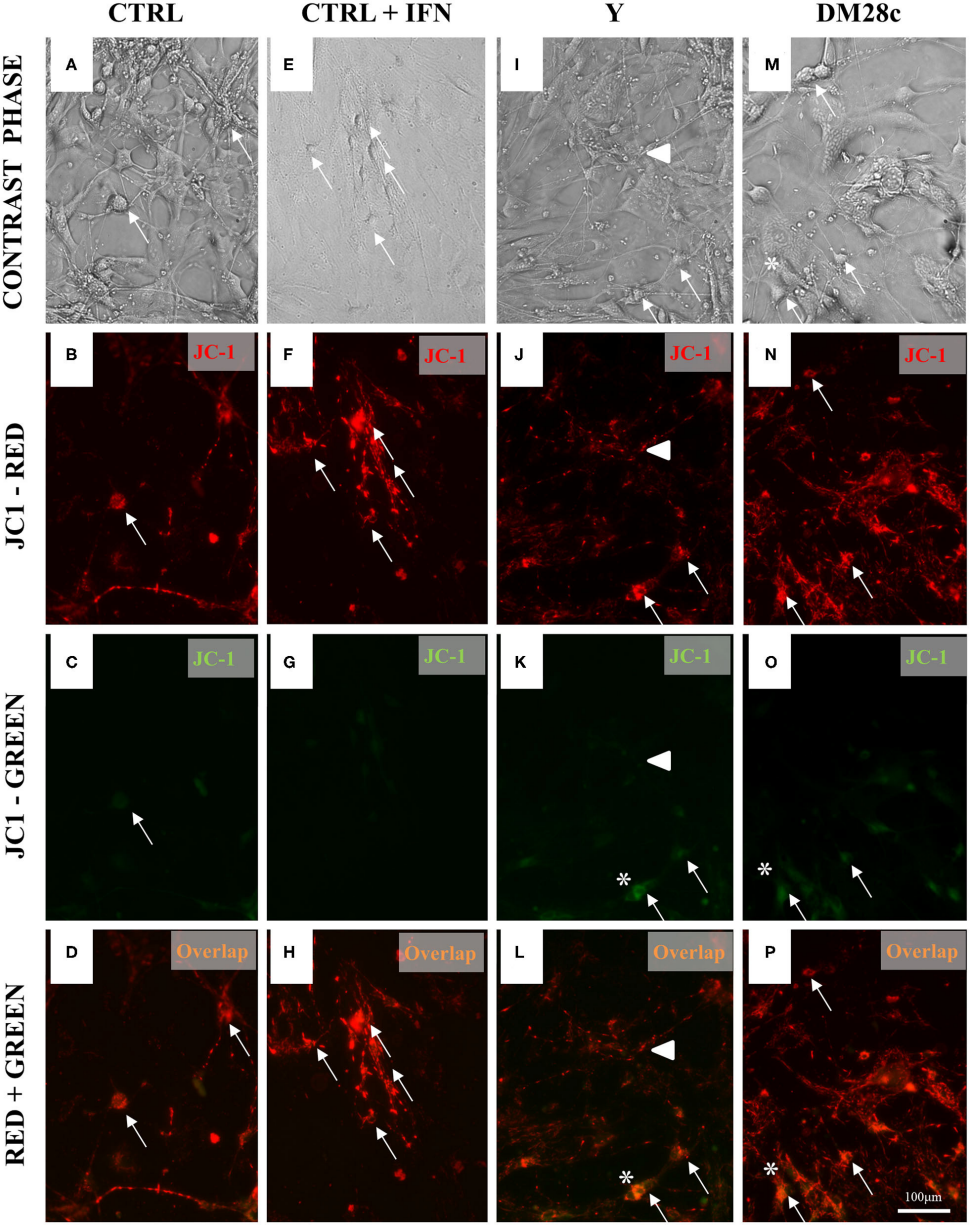
Increased mitochondrial depolarization in myenteric neurons infected with the Y and Dm28c strains of *T. cruzi*. Neurons removed from the myenteric plexus were dissociated, plated, and infected or not with *T. cruzi* strains Y and Dm28c trypomastigotes for 72 h. Then, the coverslips were washed and exposed to JC-1 to evaluate mitochondrial membrane potential *(see Methods)*. **(A–D)** Control (CTRL); **(E–H)** CTRL + IFNγ (IFN); **(I–L)** Infected with Y strain; **(M–P)** Infected with the DM28c strain. Living cells were detected by green [**(C,G,K,O)**; JC-1-monomers at the cytosol] and red fluorescence [**(B,F,J,N)**; mitochondrial matrix JC-1-aggregates] using fluorescence microscopy (Nikon Eclipse TE 200) **(A,E,I,M)** and correlated with the respective phase-contrast images **(D,H,L,P)**. Scale bar: 100 μm. 20X objective. **(C–H)** CTRL + IFNγ cultures indicating preservation of mitochondrial membrane; **(K–P)** Infected cultures. Green fluorescence increase indicating loss of mitochondrial membrane function, especially in neuronal cells (*), sometimes visibly infected (arrows), and in varicosities (arrowhead).

**Figure 7:**
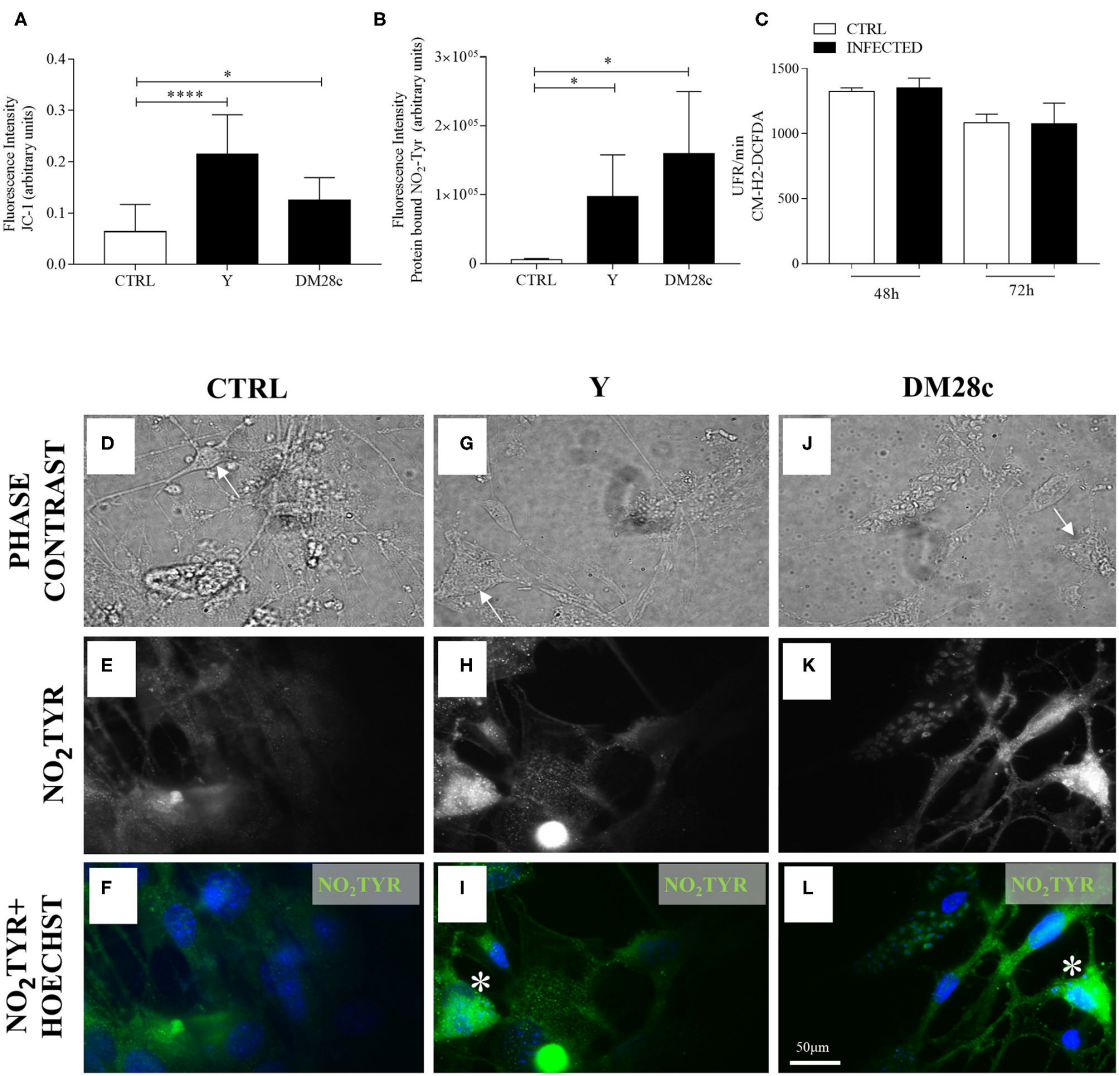
Quantification of mitochondrial function (JC-1), nitrotyrosination, and ROS production in primary enteric neuronal cultures. Neurons removed from the myenteric plexus were dissociated, plated, and infected or not with *T. cruzi* strains Y and Dm28c trypomastigotes and followed up for 72 h. **(A)** Quantification of fluorescence intensity for JC-1. *n* = 6. Data are representative of three independent experiments in triplicate coverslips **(B)** Quantification of anti-3-NO_2_-tyrosine antibody (NO_2_TYR); **(C)** Fluorescence (in relative fluorescence units, RFU) of the oxidized CM-H_2_DCFDA probe. *n* = 4. Data are representative of two independent experiments in duplicate coverslips. Phase contrast images of **(D)** Control (CTRL); **(G)** Infected with the Y strain; **(J)** infected with the Dm28c strain. **(E–L)** Immunofluorescence for NO_2_TYR. After fixing the coverslips in a 4% buffered paraformaldehyde solution, they were incubated with anti-3-NO_2_-tyrosine (green) and photographed under a fluorescence microscope (Nikon Eclipse TE 200). Cell nuclei were identified using the Hoechst marker (blue). Scale bar: 50 μm. 40X Objective. Student *t*-test. Difference in relation to the control group, *P* < 0.05 (*) *P* < 0.0001 (****). Data are shown as mean and standard deviation (SD). Arrows indicate neuronal bodies. Asterisk indicates strong immunofluorescence of nitrotyrosine in neurons.

To understand how mitochondrial abnormalities in the ENS relates to enteric neurodegeneration and to test whether infection with *T. cruzi* is associated with the depolarization of the mitochondrial inner membrane, we used JC-1, a cationic dye that exhibits a potential-dependent accumulation in mitochondria, indicated by a fluorescence emission shift. JC-1 fluorescence was detected in primary cultures of myenteric neurons infected with trypomastigotes of the Y and DM28c *T. cruzi* strains, suggesting that infection impaired mitochondrial function as early as 48 h.p.i., when parasites were detected inside the cells. As shown in Figure 6, mitochondrial depolarization is indicated by a decrease in red/green fluorescence intensity. Non-infected cultures (Figures 6A–D) and IFNγ-treated cultures (Figures 6E–H) evidenced a slight (green) color shift, indicating low levels of mitochondrial depolarization. Infection of primary neuronal cultures with both Y (Figures 6I–L) and DM28c (Figures 6M–P) strains induced an increase in green fluorescence (Figures 6K,O), which is suggestive of significant mitochondrial membrane depolarization. This was quantified in the neuronal cell bodies, by determining the green/red fluorescence ratio (Figure 7A).

### Detection of 3-NO_2_-Tyrosine in Primary Myenteric Neuronal Cultures Infected With *T. cruzi*

Because NO production and mitochondrial depolarization were enhanced by *T. cruzi* infection, we searched for the presence of 3-NO_2_-tyrosine in proteins of primary myenteric neuronal cultures infected with trypomastigotes of the Y and DM28c strains at 72 h.p.i. Figure 7 shows damaged cells of infected cultures (some of which preserving their neuronal morphology; arrows), which were more intensely labeled with anti-3-NO-tyrosine antibody (Figures 7G–L) than neurons of non-infected cultures (Figures 7D–F). Additional evidence for neuronal death in infected neuronal cultures is shown in Figure 7B, where fluorescence intensity was measured in cells recognized by phase contrast to present neuronal morphology. Infection of primary myenteric neuronal cultures with Y and DM28c strains induced a significant increase in the neuronal levels of nitrotyrosine compared to controls (*P* < 0.05).

### MnSOD2 Expression Is (Mostly) Increased in Neuronal Cells of Cultures Infected With *T. cruzi*

**Figure 8:**
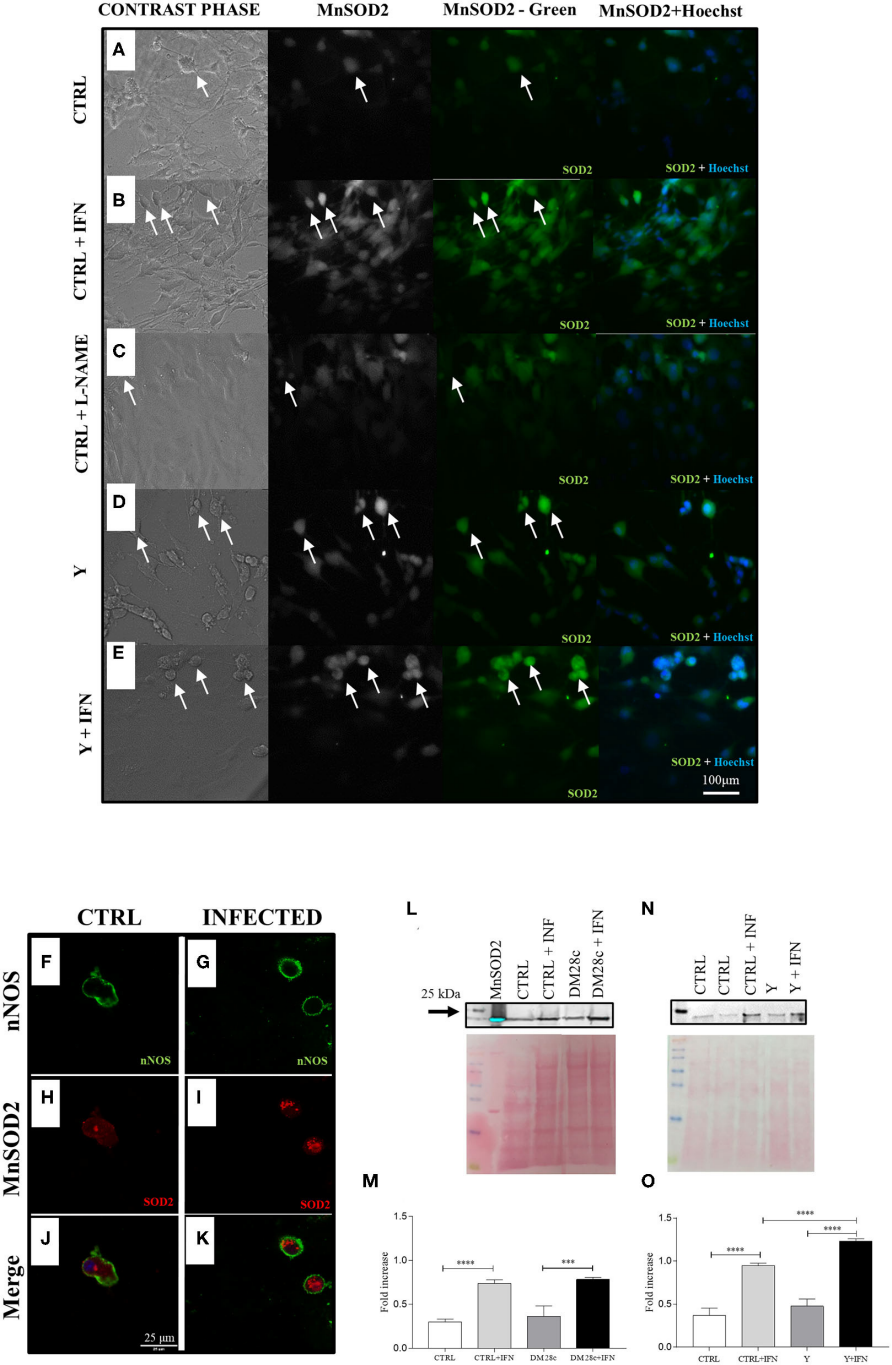
nNOS neurons infected with strains Y and Dm28c show greater expression of MnSOD2. Neurons removed from the myenteric plexus were dissociated, plated, and infected or not with *T. cruzi* strains Y and Dm28c trypomastigotes, followed up for 72 h and had their expression levels of mitochondrial MnSOD2 analyzed. **(A)** Control (CTRL); **(B)**, CTRL + interferon γ (IFN); **(C)**, Control (CTRL) + N(ω)-nitro-L-arginine methyl ester (L-NAME); **(D)** Infected with Y strain (Y); **(E)** Infected with Y strain + interferon γ (Y + IFN). After treatments, the coverslips were fixed with 4% paraformaldehyde and incubated with anti-MnSOD2 (green) and photographed under a fluorescence microscope (Nikon Eclipse TE 200). Cell nuclei were identified using the Hoechst marker (blue); **(D,E)** The cells with the highest fluorescence intensity for anti-MnSOD2 were those presenting neuronal morphology and infected as well as those activated with IFN; **(F–K)** Coverslips were double-labeled with anti-nNOS (green) and anti-MnSOD2 (red) and photographed with the LSM 880 confocal microscope (ZEISS, Germany); Panels **(F)** nNOS CTRL;**(G)** nNOS Y strain; **(H)** CTRL MnSOD2; **(I)** Y strain MnSOD2; **(J)** Merge CTRL; **(K)** Infected nNOS neurons (presented higher MnSOD2 staining when compared to control; **(J)** infected nNOS neurons present a higher intensity for MnSOD2 when compared to their respective control [**(H)**, CTRL, **(I)**, Y infected); Panels **(J)** CTRL and **(K)** Y strain-infected neurons show double labeling for nNOS and MnSOD2; **(L–O)** Western Blot with the anti-MnSOD2 antibody. To this end, primary cultures of enteric neurons were trypsinized and prepared for Western Blot as described in the methods section. The panels show greater expression of MnSOD2 in the cultures infected with both Y and Dm28c strains when the cells were activated using IFN. Scale bar: 100 μm. 20X lens. *n* = 4. Representative of two independent experiments duplicate coverslips. **(M,O)** quantification of MnSOD2 expression. Statistical analysis: ANOVA one-way with Tukey's *post hoc* tests. Difference between groups, *P* < 0.001 (***); *P* < 0.0001 (****). Data are shown as mean and and standard deviation (SD). White arrows indicate cells immunostained with anti-MnSOD2 and that present neuronal morphology.

We also evaluated MnSOD2 expression in primary myenteric neuronal cultures. In Figures 8A–E, the paired phase contrast and anti-MnSOD2 fluorescence images are shown for both control and Y strain infected neuronal cultures. The expression of MnSOD2 in neurons, identified by its morphology (arrows), was more intense in Y strain infected, and Y strain infected + INFγ- treated cultures (Figures 8D,E) than in non-infected cultures (Figures 8A–C).

Confocal microscopy also confirmed the nitrergic phenotype of enteric neurons by double immunofluorescence of nNOS (green; Figures 8F,G) and MnSOD2 (red; Figures 8H,I), which presented higher expression in neuronal bodies of infected than non-infected cultures (merged; Figures 8J,K). In addition, western blot analysis of proteins extracted from these cultures also indicated the presence of higher MnSOD2 content for cultures infected with Y or DM28c strains (Figures 8L–O).

## Discussion

One of the most challenging questions of CD is how *T. cruzi* leads to the manifestation of chronic intestinal lesions decades after infection and thus, when parasites are rare (Zhang and Tarleton, 1999; Tarleton, 2001; Benvenuti et al., 2008). The damage of myenteric neurons characterizes the chronic phase of the intestinal CD (Adad et al., 2013; Jabari et al., 2014). However, as the parasitism is very low in the lesions of digestive Chagas disease (da Silveira et al., 2005; Wesley et al., 2019), it has been suggested that chronic neuronal destruction might be due to the immune response that follows the infection, as proposed for heart lesions (Teixeira et al., 2011). It is not possible to determine when and how exactly this denervation occurs during the asymptomatic 20–30 years that follow the post-acute phase in humans. Indeed, most human studies were based on small, limited samples of enlarged intestine walls of surgically removed megacolon, obtained in the late stages of CD. Thus, it remains important to know the timeline of the structural injuries at the intestine, in order to plan effective interventions to prevent morbidity associated with its late manifestations. The present study was designed to reveal the temporal changes of the intestine throughout the development of the megacolon in a pre-established murine long-term model of *T. cruzi* infection developed in our laboratory (Campos et al., 2016).

Based on human and murine experimental data, it has been hypothesized that 50–80% of ganglionic denervation could be associated with the development of intestine's motility symptoms in humans (Koberle, 1968; Adad et al., 1991, 2001, 2013; Maifrino et al., 1999; Jabari et al., 2012). Here we detected ~40% of myenteric neuron loss very early in the course of the disease during the acute phase (11 d.p.i.). The consequences of the parasitism in ganglionic neuronal death were severe since, by 11 d.p.i, we detected a significant neuronal depopulation, which did not increase afterward (Figure 2K), together with the presence of residual foci of inflammatory cells in the intestine's wall. This observation suggests that the most important mechanisms of neuronal damage operate early on. Besides, we observed that, at 3 m.p.i., when there is a slight increase of PGP 9.5 staining, the infected curve was decreased along the timepoints compared to the control curve. This may indicate that, despite a possible degree of neuronal staining recovery after the acute events, the early damage has important consequences for the management of CD. We also found a progressive loss of nerve termination structures during the development of megacolon. This loss was associated with a continuous, albeit slight, inflammatory process. Indeed, the decrease in in the internal muscle layer's axonal profile was present as early as 3 m.p.i. (subacute phase) and continued up to 15 m.p.i. (chronic phase). At 3 m.p.i., only low levels of parasite DNA were detected, and the intestine wall was mostly cleared of parasitism. It is possible that the progressive absolute and relative axonal loss (7–15 m.p.i.; Figure 2M), together with the progressive hypertrophy of smooth muscle cells, which was observed at the chronic time points (Figure 3N), impact muscle wall contractility. Indeed, we noticed a decay in the intramuscular innervation of non-infected mice along time (Figure 3N). The significance of our data is reinforced by previous observations indicating that intrinsic and extrinsic innervation of the ENS deteriorates with age (Phillips and Powley, 2007; Sun et al., 2018).

Megacolon, a structural sign associated with various gastrointestinal disorders, is named after an irreversible dilation of a colonic segment. Lengthening and dilation of the colon are an important characteristic of advanced intestinal CD's megasyndromes. In our model, at 15 m.p.i., chronically infected animals presented a larger perimeter of the circumference of distal colon (mean = 11.060, SD 5.8 mm than their matched uninfected controls (mean = 8.777, SD 1.7 mm), although there was no statistical difference (*n* = 10, *p* > 0.4, data not showed). We speculate that only discrete, segmental portions of the colon become dilated, accompanying the segmental aspect of the lesions that we described using microscopy. Although pathological anatomy does not seem to be an adequate method for studying the lengthening and dilation of the rectosigmoid (Adad, 1997; Castro et al., 2012) this work follows a previous study, in which we established the increase of the model's intestinal length (dolichocolon) (Campos et al., 2016).

According to Lopes et al. (2013), who examined the longest radiological series of chagasic patients from endemic areas in Brazil and the Andes, the megacolon mostly starts with the lengthening of the sigmoid associated with an increase in the colon's diameter, at advanced stages of the disease. As shown for the esophagus (de Oliveira et al., 1995), colonic motility may reflect functional abnormalities in a stage preceding colon dilation (Santos et al., 2000). Studies using pharmacological tests (Meneghelli et al., 1983; Vieira et al., 1996) or manometry (Habr-Gama et al., 1970; Meneghelli et al., 1982) have shown the presence of neural and motor abnormalities in non-dilated colons. However, even in cases of advanced megacolon, sigmoid and rectum calibers may remain normal. In our model, elongation, hypertrophy, and denervation of the colon may precede the establishment of dilation.

Here we expanded upon our previous findings (Campos et al., 2016) by studying intermediate time intervals and show that the progressive hypertrophy of smooth muscle cells and the consequent increase of intestinal wall thickness start from 7 to 12 m.p.i., respectively. These data indicate that one of the most characteristic structural changes of the megacolon becomes well-established by 12 m.p.i., in an asymptomatic animal, similar to the human CD. Moreover, considering the observed progressive deposition of interstitial connective tissue (Masson's Trichrome) in the submucosa and muscular layer of infected animals (Supplementary Figures 1G–L, arrows), one can hypothesize that megacolon and its symptoms may be related to the cumulative impact of chronic inflammation and denervation in the intestinal smooth muscle layer.

We also noticed that well-preserved integral ganglia occur side by side with lesioned ones. Our findings challenge previous proposals that ganglionic neuronal depopulation is *per se* the cause of dysmotility and megacolon (González Cappa et al., 1987; Adad et al., 2001, 2013; Jabari et al., 2012). We found that these alterations take place early in the acute phase, even though the symptoms and pathological findings manifest decades later, probably resulting from cumulative damage to neurons and other elements of the intestinal wall as well as progressive functional impairment, thus explaining some contradictory findings (Ribeiro et al., 1998; Meneghelli, 1999, 2004; Adad et al., 2001; Da Silveira et al., 2008; Jabari et al., 2012, 2013). We observed a progressive loss of α-actin in some cells of the smooth muscle's inner layer, *in vivo*, and a qualitative and quantitative decrease and irregularity of α-actin expression *in vitro*. These changes can be interpreted as an additional factor underlying intestinal dysfunction along with the increase in the thickness of the muscular *propria*, the significant ganglionic neuronal loss, and the progressive intramuscular denervation reported herein.

As the acute inflammation changed to a pattern of chronic and sustained mononuclear inflammation, we quantified the phenotype of mononuclear cells in the inflammatory infiltrate of the muscular *propria* (Supplementary Figures 1A–C), both at the acute and chronic phases. Similar to the findings reported for the human cardiac chronic disease (Higuchi et al., 1997), mononuclear CD4+ cells increased significantly at the acute phase, whereas CD8+ cells increased at the chronic phase. A role for CD8+ cells in *T. cruzi*-dependent mechanisms involved in neuromyopathic damage was observed in lymphocyte proliferative assays against skeletal muscle, sciatic nerve, and spinal cord homogenates (Mirkin et al., 1994, 1997), and need further investigation in our *in vivo* chronic model. It is also possible that immunoregulatory mechanisms may play a role in controlling the production of inflammatory and anti-inflammatory cytokines in our chronic model, since most mononuclear cells remained non-stained by anti-CD4 and anti-CD8 antibodies (Supplementary Figures 1A,B). Interestingly, we observed that chronic inflammatory infiltrates irregularly distributed in the intestinal wall were mostly located inside the muscular layers, and only segmentally in the myenteric ganglia. This might explain the progressive intramuscular axonal loss throughout the development of the megacolon, even when ganglionic neuronal loss does not increase along time.

Because an adaptive IFNγ-dependent immune response controls the tissue parasitism, a narrower profile of chemokines is produced in the chronic inflammatory milieu of the heart (Rodrigues et al., 2009). The same probably occurs in the intestine. We found parasite DNA throughout the acute and the chronic (up to 15 m.p.i.) phases, although there was an abrupt drop in parasite's DNA at the end of the acute phase. Indeed, several studies have now established the link between the presence of parasite antigens or DNA and the inflammatory infiltrates observed in the hearts and intestines of chronic chagasic patients (Higuchi et al., 1993; Vago et al., 1996; Soares et al., 2001; Tarleton, 2001; Benvenuti et al., 2008; Vazquez et al., 2015). However, since parasitism is poorly correlated with the degree of inflammation, it is possible that other mechanisms might amplify *T. cruzi*-infection-driven inflammation, chemokine production, and pathogenesis (Leon and Engman, 2001; Teixeira et al., 2002). The investigation of the effect of *T. cruzi* infection in primary myenteric neuronal cultures up to 72 h.p.i. (Figures 4–6) complemented our *in vivo* studies. We found that neuronal bodies and the neurite network of infected primary myenteric neuronal cultures were damaged and that β-tubulin III immunostaining significantly decreased in infected neurons. The aspect of the infected cultures mimicked the *in vivo* tissue changes at the acute phase that are associated with the parasite-induced degenerative changes of enteric ganglia and muscular *propria* (Arantes et al., 2004; Campos et al., 2016).

There is scarce information on the invasion of neuronal cells and its pathogenic consequences to the disease, despite the vast literature covering diverse aspects of host-parasite interaction in several mammalian and transformed cell types (Andrews et al., 1987). A systematic electron microscopy investigation conducted by Tafuri and collaborators detected amastigotes in glia cells, capsular fibroblasts, ganglion interstitium, and in the connective tissue close to the ganglion, but not in enteric neurons (Tafuri et al., 1971). Interestingly, direct neuronal *T. cruzi* invasion was never deemed as the cause of ganglionic lesions, which were solely attributed to the inflammation-induced swelling and degenerative changes of ganglia components. Ours is the first study to investigate infection in primary cultures of myenteric neurons, thus opening a new avenue for studies on parasite-neuron interaction. Our study is also the first to detect parasite invasion and proliferation, not only in smooth muscle cells but also in primary myenteric neurons *in vitro* (Figure 4).

The predominant neuronal phenotype found in the primary cultures of myenteric neurons studied herein were nitrergic neurons. Most nNOS+ neurons presented a small number of parasites inside their neuronal bodies at 72 h.p.i. (Figure 4P). Some non-nitrergic neurons were infected. Parasite replication does not occur in neurons as frequently as in other cells; thus, it is possible that the neurons do not die only due to direct parasitism. Parasites were easily seen by H&E and anti-*T. cruzi* immunostaining inside glia cells and smooth muscle cells close to the enteric neurons. Loss of neuronal morphology or selective neuronal vulnerability to oxidative stress (Wang and Michaelis, 2010; Rivera et al., 2011) may be the reasons why previous *in vivo* and *in vitro* studies failed to detect neuronal invasion. The vulnerability of enteric neurons in CD was never studied before, and the resistance of superior cervical ganglion neurons to *T. cruzi* invasion (de Almeida-Leite et al., 2014) also needs further addressing. The *in vivo* approach adopted herein should allow a better evaluation of the role of neuronal invasion in neuronal death.

The neuronal death observed during the acute phase was, at least partially, the consequence of RNS production with protein NO_2_-tyrosine modifications, especially in nitregic neurons. While NO normally functions as a physiological neuronal mediator, excess production of NO can lead to neuronal injury (Wang and Michaelis, 2010). As free radicals are inherently reactive, NO causes cellular toxicity by damaging critical metabolic enzymes and reacts with radical superoxide to form superoxide, is a potent oxidant (Bredt, 1999). Through these mechanisms NO appears to play a pivotal role in the pathophysiology of intestinal damage associated with CD.

IFNγ-stimulation of non-infected primary cultures of myenteric neurons and *T. cruzi* infection increased DAF fluorescence in myenteric neuronal cells. Also, infected myenteric neurons exhibited higher oxidation of DAF than infected glia, smooth muscle, and non-infected neurons in the same cultures. These data indicate, for the first time, that an increase in NO production was elicited by *T. cruzi* infection (Y and DM28c strains). Moreover, in our model, DAF-FM-positive cells correlated well with neuronal morphology (Figure 5) and may implicate nitrergic neurons as targets for direct *T. cruzi* invasion (Figures 5E,F). Cytokines, such as IFNγ (Figures 5I,J), are probably produced in the proximity of ganglionic neurons and its varicosities by glia and smooth muscle cells. We also observed DAF oxidation around amastigotes inside neuronal and non-neuronal cells present in the cultures. The increase of DAF oxidation has been previously evaluated *in vitro* in isolated *T. cruzi*. These parasites produce RNS when treated with an anti-tumoral combination of vitamins K3 and C (Desoti et al., 2015), and with anti-*T. cruzi* (1,3,4-Thiadiazole) derivatives (Desoti et al., 2015; Martins et al., 2016). Importantly, our study is the first to suggest the production of RNS by parasites inside the neurons, a finding that is intriguing and deserves further investigation.

NOS neurons face the additional problem that nNOS activation depends upon the binding of Ca^2+^-calmodulin, and therefore, NO generation is stimulated by an increase of intracellular Ca^2+^ (Cho et al., 1992; Hemmens and Mayer, 1998; Forstermann and Sessa, 2012). This is hypothesized to produce an excess of NO that is further exacerbated by the peroxynitrite formed when NO reacts with superoxide radicals (Voukali et al., 2011). In the intestine, the nNOS selectively concentrated in axon varicosities of myenteric neurons (Bredt et al., 1990; Bredt, 1999) may be related to the progressive decrease of axonal density observed in our model.

Because *T. cruzi* invasion of most mammalian cells mobilizes Ca^2+^ (Burleigh and Andrews, 1998), we used Ionomicyn, a Ca^2+^ ionophore in infected primary cultures of myenteric neurons to test if the same mechanism was taking place in our cultures. With this strategy, parasited and non-parasited neurons in the same preparation were accessed simultaneously as intra-experimental controls. Y-strain invaded neurons treated with ionomycin showed higher DAF oxidation (Figures 5A,B,L) than non-invaded neurons (Figures 5I,J). Our results imply that the enhanced neuronal intracellular Ca^2+^ concentration triggered by *T. cruzi* infection and glia-released IFNγ in the proximity of ganglionic neurons and its varicosities, may lead to increased production of RNS.

We tested if NO, produced by NOS in the neuronal cytoplasm, and ROS from intracellular or extracellular sources could be reacting within nitrergic neurons, exposing them to a higher degree of cytotoxic cell damage compared with other neurons. However, using the CM-H2DCFDA probe, we did not detect differences in ROS production between infected and non-infected cultures at 48 and 72 h.p.i. (Figure 7C). The irregularity of the neuronal culture monolayer and the threshold of detectable ROS production by the fluorimeter probably limited our assays. We believe that the single-cell fluorescence imaging approach (Gonzalez and Salido, 2016) would have detected the probe inside cells.

We also found evidence to implicate protein tyrosine nitration in infected cultures (Y and DM28c strains) with the acute neuronal death observed in CD. The fluorescence intensity of 3-NO_2_-tyrosine in the infected cultures was significantly increased in parasited neurons compared to non-parasited neurons present in the same cultures (intra-experimental controls). 3-NO_2_-tyrosine constitutes a footprint left by the reactions of NO-derived oxidants in protein tyrosine residues to form 3-nitrotyrosine. Moreover, protein tyrosine nitration can cause structural and functional changes, which may be of pathophysiological relevance for human disease. Indeed, the formation of 3-NO_2_-tyrosine *in vivo* occurs in diverse pathologic conditions, and this protein is thought to be a specific marker of oxidative damage mediated by peroxynitrite (Ahsan, 2013). Much of •NO-mediated pathogenicity depends on the formation of secondary intermediates such as peroxynitrite (ONOOH/ONOO-; pKa = 6.8) that is typically more reactive and toxic than •NO (Radi, 2013; Batthyány et al., 2017).

As mitochondrial damage is implicated in neurodegenerative diseases (Cenini et al., 2019), we investigated the role of mitochondrial deficits in ENS neurodegeneration in infected primary cultures of myenteric neurons by checking for shifts in JC-1fluorescence (Figure 6). JC-1 is more specific for mitochondrial vs. plasma membrane potential in its response to depolarization than other cationic dyes (Ankarcrona et al., 1995; White and Reynolds, 1996; Kulkarni et al., 1998). The ratio of green to red fluorescence is dependent only on the membrane potential and not on other factors that may influence single-component fluorescence signals. Therefore, fluorescence ratio detection allows researchers to make comparative measurements of membrane potential. We found that infection using both *T. cruzi* strains tested (Y and DM28c) was associated with depolarization of the mitochondrial inner membrane, which indicates mitochondrial function impairment, as early as 48 h.p.i., when parasites were detected inside the cells. Interestingly, mitochondrial defects in Tfam-ENSKO mice differentially affected specific subpopulations of enteric neurons, such as nitrergic inhibitory neurons, resulting in an imbalance of inhibitory and excitatory neurons (Viader et al., 2011). This observation may help explain the mitochondrial deficits and neurodegenerative changes in infected nNOS neurons detected in our assays.

Greater expression of MnSOD2 is a defense response to ROS induced in *T. cruzi* invaded cardiomyocytes (Estrada et al., 2018). MnSOD2 protects cells from the deleterious effects of the overproduction of NO and peroxynitrite (Candas and Li, 2014). To confirm that oxidative stress operates in the *T. cruzi*-induced neuronal damage *in vitro*, we investigated the expression of MnSOD2 in nitrergic neurons. We detected increased MnSOD2 expression in neurons of infected cultures of both *T. cruzi* strains (Y and DM28c) by fluorescence quantification and Western blot. Confocal microscopy evidenced higher expression of MnSOD in nNOS+ neuronal bodies. In this scenario, where host-derived oxidant mediators are actively generated, the antioxidant arsenal of *T. cruzi* may become decisive for parasite survival and persistence (Ferreira et al., 1997; Piacenza et al., 2009a,b; Radi, 2018). The hypothesis that enzymes of the parasite antioxidant arsenal may represent novel virulence factors involved in the establishment of the disease needs further exploitation in neuronal systems.

Altogether, our results reinforced that oxidative stress has neurotoxic consequences in *T.cruzi* infection. The selective vulnerability of neurons occurs in conditions involving a high demand of ROS/RNS. Signaling molecules, chronic inflammation, and calcium deregulation in vulnerable neurons (Wang and Michaelis, 2010), could explain our results.

Despite many decades of research on CD, the factors that steer chronic CD from an asymptomatic state to clinical onset remain unclear. Success in translating the vast information available about CD into concrete applications are scarce, particularly in the case of intestinal chronic CD pathology. Our model of intestinal CD conciliates the existing pathogenesis hypotheses of megacolon. We propose that long-term intestinal remodeling (axonal and synaptic damages as well as and smooth muscle degenerative changes) is the basis for the megacolon and its related functional disorders. Our findings suggest that prevention of oxidative stress may help to treat this highly morbid condition in newly infected individuals (acute and subacute phases). An integrative comprehension of acute and chronic phases of the *T. cruzi* infection should help drive research to make a real difference for the millions of people affected by this neglected disease.

## Data Availability Statement

The raw data supporting the conclusions of this article will be made available by the authors, without undue reservation.

## Ethics Statement

The animal study was reviewed and approved by the Institutional Committee for Animal Ethics of UFMG (CEUA/UFMG–Licenses 262/2016 and 25/2018). This study was carried out in strict accordance with the recommendations of the Guide for the Care and Use of Laboratory Animals of the Brazilian National Council of Animal Experimentation (http://www.cobea.org.br/) and Federal Law 11.794 (October 8, 2008).

## Author Contributions

MR, LP, and RA: conceptualization. MR, LP, MM, and RA: data curation. MR, MM, and RA: formal analysis. RA, LA, and RR: funding acquisition. MR, SB, LP, RR, MB, AM, and AO: methodology. RA and MB: resources. RA, MB, LA, LP, and RR: supervision. MR, MB, LA, LP, and RA: validation. MR, SB, RA, MB, LA, and LP: visualization. MR, RA, and LA: writing–original draft. RA, LP, and LA: writing–review and editing. All authors contributed to the article and approved the submitted version.

## Conflict of Interest

The authors declare that the research was conducted in the absence of any commercial or financial relationships that could be construed as a potential conflict of interest.

## Supplementary Material

Supplementary Figure 1 Histopathological aspects of the colonic changes induced by the infection with the Y strain of *T. cruzi*. **(A)** CD4+ and **(B)** CD8+ cells observed in muscularis propria of chronic infected animals (15 m.p.i.) were quantified and expressed as **(C)** percentage of imunophenotyped (CD4+ and CD8+) cells/total cells of control and infected mice at acute (IAP, 11 d.p.i.) and chronic phase (ICP15, 15 m.p.i.). The percentage of CD4 + cells / total cells was higher for the infected groups when compared to the control groups (CTRL). The percentage of CD8 + cells / total cells decreased in IAP when compared to CTRL (CAP) animals, but increased in ICP15 mice (*P* = 0.07); **(D–H)** Histopathological aspects observed in colon samples of infected animals stained with HE where **(D)** CAP, **(E)** IAP, **(F)** ICP3, **(G)** ICP7, **(H)** ICP12, and **(I)** ICP15. Inflammatory foci were found scattered throughout the colon wall, at all times of the experiment. Notice that inflammatory cells in **(E)** (IAP) and **(F)** (ICP3) are associated with the vacuolization of parenchyma cells and hyper-eosinophilia. The infiltrate surrounds the enteric ganglia and sometimes blur its limits (inset, dashed line). Arrowhead indicates inflammatory infiltrate. **(J)** CTRL, **(K)** IAP, **(L)** ICP3, **(M)** ICP7, **(N)** ICP12, and **(O)** ICP15 Masson's trichome stains the connective tissue in blue. Reorganization and increased production of collagen fibbers throughout the infection. Thin arrows indicate interstitial and periganglionic collagen fibers, large arrows indicate collagen fibbers in the submucosal plexus. Scale Bar: 3 μm. 20X objective. Statistical analysis: ANOVA one-way with Student-Newman-Keuls *post hoc* tests. Difference in relation to the control group, *P* ≤ 0.05 (^*^) (*n* = 5 mice). Representative of two independent experiments. Data are shown as mean and and standard deviation (SD).

## Abbreviations

AP: acute phase
BZ: benznidazole
Ca^2+^: calcium
CAP: control acute phase
CCP: control chronic phase
CD: Chagas' disease
CM-H_2_DCFDA: 5-(and-6)-chloromethyl-2', 7'-dichlorodihydrofluorescein diacetate, acetyl ester
CP: chronic phase
CTRL: control
CV: coefficient of variation
DAF-FM: 4-Amino-5-Methylamino-2', 7'-Difluorofluorescein Diacetate
d.p.i.: days post-infection
ENS: enteric nervous system
h.p.i.: hours post-infection
IAP: infected acute phase
ICB: Institute of Biological Science
ICP: infected chronic phase
JC-1: J-aggregate-forming lipophilic cation
L-NAME: N(ω)-nitro-L-arginine methyl ester
MnSOD2: manganese superoxide dismutase 2
m.p.i.: months post-infection
NO: nitric oxide
3-NO_2_Tyr: Nitrotyrosine
nNOS: neuronal nitric oxide synthase
PGP 9.5: protein gene product 9.5
RNS: reactive nitrogen species
ROS: reactive oxygen species
*T. cruzi*: *Trypanosoma cruzi*.

## References

[B1] Adad S. J. (1997). Contribution to the study of pathology and pathogenesis of chagasic mecolon. Rev. Soc. Bras. Med. Trop. 30, 79–81. 10.1590/S0037-86821997000100018

[B2] Adad S. J. Andrade D. C. Lopes E. R. Chapadeiro E. (1991). Pathological anatomy of chagasic megaesophagus. Rev. Inst. Med. Trop. Saõ Paulo 33, 443–450. 10.1590/S0036-46651991000600004 1844974

[B3] Adad S. J. Cançado C. G. Etchebehere R. M. Teixeira V. P. Gomes U. A. Chapadeiro E.. (2001). Neuron count reevaluation in the myenteric plexus of chagasic megacolon after morphometric neuron analysis. Virchows Arch. 438, 254–258. 10.1007/s004280000319 11315622

[B4] Adad S. J. Silva G. B. E. Jammal A. A. (2013). The development of chagasic megacolon requires severe denervation and the reduction in interstitial cells of Cajal number might be a contributing factor. Virchows Arch. 462:127. 10.1007/s00428-012-1349-1 23224119

[B5] Ahsan H. (2013). 3-Nitrotyrosine: A biomarker of nitrogen free radical species modified proteins in systemic autoimmunogenic conditions. Hum. Immunol. 74, 1392–9. 10.1016/j.humimm.2013.06.009 23777924

[B6] Aliberti J. C. Machado F. S. Souto J. T. Campanelli A. P. Teixeira M. M. Gazzinelli R. T.. (1999). Beta-chemokines enhance parasite uptake and promote nitric oxide-dependent microbiostatic activity in murine inflammatory macrophages infected with *Trypanosoma cruzi*. Infect. Immun. 67, 4819–4826. 10.1128/IAI.67.9.4819-4826.1999 10456936 PMC96814

[B7] Almeida-Leite C. M. Galvão L. M. Afonso L. C. Cunha F. Q. Arantes R. M. E. (2007). Interferon-gamma induced nitric oxide mediates *in vitro* neuronal damage by *Trypanosoma cruzi*-infected macrophages. Neurobiol. Dis. 25, 170–178. 10.1016/j.nbd.2006.09.003 17056264

[B8] Andrews N. W. Hong K. S. Robbins E. S. Nussenzweig V. (1987). Stage-specific surface antigens expressed during the morphogenesis of vertebrate forms of *Trypanosoma cruzi*. Exp. Parasitol. 64, 474–484. 10.1016/0014-4894(87)90062-2 3315736

[B9] Ankarcrona M. Dypbukt J. M. Bonfoco E. Zhivotovsky B. Orrenius S. Lipton S. A. (1995). Glutamate-induced neuronal death: a succession of necrosis or apoptosis depending on mitochondrial function. Neuron 15, 961–973. 10.1016/0896-6273(95)90186-8 7576644

[B10] Arantes R. Lourenssen S. Machado C. Blennerhassett M. (2000). Early damage of sympathetic neurons after co-culture with macrophages: a model of neuronal injury *in vitro*. Neuroreport 11, 177–181. 10.1097/00001756-200001170-00035 10683853

[B11] Arantes R. M. Nogueira A. M. (1997). Distribution of enteroglucagon- and peptide YY-immunoreactive cells in the intestinal mucosa of germ-free and conventional mice. Cell Tissue Res. 290, 61–69. 10.1007/s004410050908 9377643

[B12] Arantes R. M. E. Marche H. H. F. Bahia M. T. Cunha F. Q. Rossi M. A. Silva J. S. (2004). Interferon-gamma-induced nitric oxide causes intrinsic intestinal denervation in *Trypanosoma cruzi*-infected mice. Am. J. Pathol. 164, 1361–1368. 10.1016/S0002-9440(10)63222-1 15039223 PMC1615344

[B13] Balcerczyk A. Soszynski M. Bartosz G. (2005). On the specificity of 4-amino-5-methylamino-2′, 7′-difluorofluorescein as a probe for nitric oxide. Free Radic. Biol. Med. 39, 327–335. 10.1016/j.freeradbiomed.2005.03.017 15993331

[B14] Batthyány C. Bartesaghi S. Mastrogiovanni M. Lima A. Demicheli V. Radi R. (2017). Tyrosine-nitrated proteins: proteomic and bioanalytical aspects. Antioxid. Redox Signal. 26, 313–328. 10.1089/ars.2016.6787 27324931 PMC5326983

[B15] Benvenuti L. A. Roggério A. Freitas H. F. G. Mansur A. J. Fiorelli A. Higuchi M. L. (2008). Chronic American trypanosomiasis: parasite persistence in endomyocardial biopsies is associated with high-grade myocarditis. Ann. Trop. Med. Parasitol. 102, 481–487. 10.1179/136485908X311740 18782487

[B16] Bereiter-Hahn J. (1975). Dimethylaminostyrylmethylpyridiniumiodine (daspmi) as a fluorescent probe for mitochondria *in situ*. Biochim Biophys Acta. 15, 1–14. 10.1016/0005-2728(76)90096-7 764877

[B17] Bonney K. M. Engman D. M. (2008). Chagas heart disease pathogenesis: one mechanism or many? Curr. Mol. Med. 8, 510–518. 10.2174/156652408785748004 18781958 PMC2859714

[B18] Braga E. M. Galvão L. M. Chiari E. Martins M. S. (1993). Difference in susceptibility to lysis between clones of the Y strain of *Trypanosoma cruzi*. Mem. Inst. Oswaldo Cruz 88, 529–34. 10.1590/S0074-02761993000400005 8139464

[B19] Bredt D. S. (1999). Endogenous nitric oxide synthesis: biological functions and pathophysiology. Free Radic. Res. 31, 577–596. 10.1080/10715769900301161 10630682

[B20] Bredt D. S. Hwang P. M. Snyder S. H. (1990). Localization of nitric oxide synthase indicating a neural role for nitric oxide. Nature 347, 768–770. 10.1038/347768a0 1700301

[B21] Brener Z. (1962). Therapeutic activity and criterion of cure on mice experimentally infected with *Trypanosoma cruzi*. Rev. Inst. Med. Trop. Saõ Paulo 4, 389–396. 14015230

[B22] Brener Z. Andrade Z. Barral-Neto M. (2000). Trypanosoma cruzi e Doença de Chagas. Rio de Janeiro: Guanabara Koogan.

[B23] Brito C. Naviliat M. Tiscornia A. C. Vuillier F. Gualco G. Dighiero G.. (1999). Peroxynitrite inhibits T lymphocyte activation and proliferation by promoting impairment of tyrosine phosphorylation and peroxynitrite-driven apoptotic death. J. Immunol. 162, 3356–3366. 10092790

[B24] Bubenheimer R. K. Brown I. A. M. Fried D. E. McClain J. L. Gulbransen B. D. (2016). Sirtuin-3 is expressed by enteric neurons but it does not play a major role in their regulation of oxidative stress. Front. Cell. Neurosci. 10:73. 10.3389/fncel.2016.00073 27047337 PMC4801875

[B25] Burgos J. M. Diez M. Vigliano C. Bisio M. Risso M. Duffy T.. (2010). Molecular identification of *Trypanosoma cruzi* discrete typing units in end-stage chronic chagas heart disease and reactivation after heart transplantation. Clin. Infect. Dis. 51, 485–495. 10.1086/655680 20645859

[B26] Burleigh B. A. Andrews N. W. (1998). Signaling and host cell invasion by *Trypanosoma cruzi*. Curr. Opin. Microbiol. 1, 461–465. 10.1016/S1369-5274(98)80066-0 10066513

[B27] Campos C. F. Cangussú S. D. Duz A. L. Cartelle C. T. Noviello Mde L. Veloso VM.. (2016). Enteric neuronal damage, intramuscular denervation and smooth muscle phenotype changes as mechanisms of chagasic megacolon: evidence from a long-term murine model of *Tripanosoma cruzi* infection. PLoS ONE 11:e0153038. 10.1371/journal.pone.0153038 27045678 PMC4821538

[B28] Candas D. Li J. J. (2014). MnSOD in oxidative stress response-potential regulation via mitochondrial protein influx. Antioxid. Redox Signal. 20, 1599–1617. 10.1089/ars.2013.5305 23581847 PMC3942709

[B29] Castro C. Hernandez E. B. Rezende J. Prata A. (2012). Occurrence of dolichocolon without megacolon in chronic chagas disease patients. Rev. Soc. Bras. Med. Trop. 45, 353–356. 10.1590/S0037-86822012000300014 22760135

[B30] Cenini G. Lloret A. Cascella R. (2019). Oxidative Stress in neurodegenerative diseases: from a mitochondrial point of view. Oxid. Med. Cell. Longev. 2019, 1–18. 10.1155/2019/2105607 31210837 PMC6532273

[B31] Cenini G. Lloret A. Cascella R. (2020). Oxidative stress and mitochondrial damage in neurodegenerative diseases: from molecular mechanisms to targeted therapies. Oxid. Med. Cell. Longev. 2020, 1–2. 10.1155/2020/1270256 32454930 PMC7222558

[B32] Cho H. J. Xie Q. W. Calaycay J. Mumford R. A. Swiderek K. M. Lee T. D.. (1992). Calmodulin is a subunit of nitric oxide synthase from macrophages. J. Exp. Med. 176, 599–604. 10.1084/jem.176.2.599 1380065 PMC2119310

[B33] Cruz J. S. Santos-Miranda A. Sales-Junior P. A. Monti-Rocha R. Campos P. P. Machado F. S.. (2016). Altered cardiomyocyte function and *Trypanosoma cruzi* persistence in chagas disease. Am. J. Trop. Med. Hyg. 94, 1028–1033. 10.4269/ajtmh.15-0255 26976879 PMC4856598

[B34] Cummings K. L. Tarleton R. L. (2003). Rapid quantitation of *Trypanosoma cruzi* in host tissue by real-time PCR. Mol. Biochem. Parasitol. 129, 53–59. 10.1016/S0166-6851(03)00093-8 12798506

[B35] Da Silveira A. Freitas M. de Oliveira E. Neto S. Luquetti A. Furness J.. (2008). Substance P and NK1 receptor expression in the enteric nervous system is related to the development of chagasic megacolon. Trans. R. Soc. Trop. Med. Hyg. 102, 1154–1156. 10.1016/j.trstmh.2008.04.043 18554673

[B36] da Silveira A. B. M. Arantes R. M. E. Vago A. R. Lemos E. M. Adad S. J. Correa-Oliveira R.. (2005). Comparative study of the presence of *Trypanosoma cruzi* kDNA, inflammation and denervation in chagasic patients with and without megaesophagus. Parasitology 131, 627–634. 10.1017/S0031182005008061 16255821

[B37] de Almeida-Leite C. M. Silva I. C. C. Galvão L. M. Arantes R. M. E. (2014). Sympathetic glial cells and macrophages develop different responses to *Trypanosoma cruzi* infection or lipopolysaccharide stimulation. Mem. Inst. Oswaldo Cruz 109, 459–465. 10.1590/0074-0276130492 25075784 PMC4155848

[B38] de Oliveira R. B. Rezende Filho J. Dantas R. O. Iazigi N. (1995). The spectrum of esophageal motor disorders in chagas' disease. Am. J. Gastroenterol. 90, 1119–1124. 7611209

[B39] De Oliveira R. B. Troncon L. E. A. Dantas R. O. Meneghelli U. G. (1998). Gastrointestinal manifestations of chagas' disease. Am. J. Gastroenterol. 93, 884–889. 10.1111/j.1572-0241.1998.270_r.x 9647012

[B40] DeFaria C. R. De Rezende J. M. Rassi A. (1988). Peripheral denervation in the various clinical forms of chagas' disease. Arq. Neuropsiquiatr. 46, 225–237. 10.1590/S0004-282X1988000300001 2851967

[B41] Desoti V. C. Lazarin-Bidóia D. Ribeiro F. M. Martins S. C. da Silva Rodrigues J. H. Ueda-Nakamura T.. (2015). The combination of vitamin K3 and vitamin C has synergic activity against forms of *Trypanosoma cruzi* through a redox imbalance process. PLoS ONE 10:e0144033. 10.1371/journal.pone.0144033 26641473 PMC4671608

[B42] Dias P. P. Capila R. F. do Couto N. F. Estrada D. Gadelha F. R. Radi R.. (2017). Cardiomyocyte oxidants production may signal to *T. cruzi* intracellular development. PLoS Negl. Trop. Dis. 11:e0005852. 10.1371/journal.pntd.0005852 28832582 PMC5584977

[B43] Ducci H. Pizzi T. (1949). Miocarditis chagásica. Rev. Med. Chil. 77, 207–209. 18127934

[B44] Dutra W. O. Menezes C. A. S. Magalhães L. M. D. Gollob K. J. (2014). Immunoregulatory networks in human chagas disease. Parasite Immunol. 36, 377–387. 10.1111/pim.12107 24611805 PMC4143493

[B45] Dutta S. Sengupta P. (2016). Men and mice: relating their ages. Life Sci. 152, 244–248. 10.1016/j.lfs.2015.10.025 26596563

[B46] Estrada D. Specker G. Martínez A. Dias P. P. Hissa B. Andrade L. O.. (2018). Cardiomyocyte diffusible redox mediators control *Trypanosoma cruzi* infection: role of parasite mitochondrial iron superoxide dismutase. Biochem. J. 475, 1235–1251. 10.1042/BCJ20170698 29438066

[B47] Ferreira M. S. Nishioka Sde A. Silvestre M. T. Borges A. S. Nunes-Araujo F. R. Rocha A.. (1997). Reactivation of chagas' disease in patients with AIDS: report of three new cases and review of the literature. Clin. Infect. Dis. 25, 1397–1400. 10.1086/516130 9431385

[B48] Flurkey K. Currer J. M. Harrison D. E. (2007). “The mouse in aging research,” in The Mouse in Biomedical Research, eds Foster H. L. Smart J. D. Fox J. G. (Burlington, MA: Elsevier), 637–672. 10.1016/B978-012369454-6/50074-1

[B49] Forstermann U. Sessa W. C. (2012). Nitric oxide synthases: regulation and function. Eur. Heart J. 33, 829–837. 10.1093/eurheartj/ehr304 21890489 PMC3345541

[B50] Garcia S. B. Paula J. S. Giovannetti G. S. Zenha F. Ramalho E. M. Zucoloto S.. (1999). Nitric oxide is involved in the lesions of the peripheral autonomic neurons observed in the acute phase of experimental *Trypanosoma cruzi* infection. Exp. Parasitol. 93, 191–197. 10.1006/expr.1999.4451 10600444

[B51] Gazzinelli R. T. Oswald I. P. Hieny S. James S. L. Sher A. (1992). The microbicidal activity of interferon-gamma-treated macrophages against *Trypanosoma cruzi* involves an L-arginine-dependent, nitrogen oxide-mediated mechanism inhibitable by interleukin-10 and transforming growth factor-beta. Eur. J. Immunol. 22, 2501–2506. 10.1002/eji.1830221006 1396957

[B52] González Cappa S. M. Sanz O. P. Muller L. A. Molina H. A. Fernández J. Rimoldi M. T.. (1987). Peripheral nervous system damage in experimental chronic chagas' disease. Am. J. Trop. Med. Hyg. 36, 41–45. 10.4269/ajtmh.1987.36.41 3101527

[B53] Gonzalez A. Salido G. M. (2016). Determination of reactive oxygen species production in pancreatic acinar cells. Pancreapedia Exocrine Pancreas Knowl. Base 1:9 10.3998/panc.2016.32

[B54] Guillén-Pernía B. Lugo-Yarbuh A. Moreno E. (2001). Dilatación del tracto digestivo de ratones infectados con *Trypanosoma cruzi* [Digestive tract dilation in mice infected with *Trypanosoma cruzi*]. Invest. Clin. 42, 195–209. 11552508

[B55] Gupta S. Bhatia V. Wen J. Wu Y. Huang M.-H. Garg N. J. (2009). *Trypanosoma cruzi* infection disturbs mitochondrial membrane potential and ROS production rate in cardiomyocytes. Free Radic. Biol. Med. 47, 1414–1421. 10.1016/j.freeradbiomed.2009.08.008 19686837 PMC2767388

[B56] Gupta S. Garg N. J. (2013). TcVac3 induced control of *Trypanosoma cruzi* infection and chronic myocarditis in mice. PLoS ONE 8:e59434 10.1371/journal.pone.0059434 23555672 PMC3608676

[B57] Habr-Gama A. Costa-Curta L. Raia A. (1970). Anatomia e fisiologia do esfíncter interno do ânus. Rev. Bras. Colo-Proctol. 3, 21–30.

[B58] Hemmens B. Mayer B. (1998). Enzymology of nitric oxide synthases. Methods Mol. Biol. 100, 1–32. 10.1385/1-59259-749-1:1 10906988

[B59] Higuchi M. D. L De Brito T. Martins Reis M. Barbosa A. Bellotti G. Pereira-Barreto A. C.. (1993). Correlation between *Trypanosoma cruzi* parasitism and myocardial inflammatory infiltrate in human chronic chagasic myocarditis: light microscopy and immunohistochemical findings. Cardiovasc. Pathol. 2, 101–106. 10.1016/1054-8807(93)90021-S 25990604

[B60] Higuchi M. D. Ries M. M. Aiello V. D. Benvenuti L. A. Gutierrez P. S. Bellotti G.. (1997). Association of an increase in CD8+ T cells with the presence of *Trypanosoma cruzi* antigens in chronic, human, chagasic myocarditis. Am. J. Trop. Med. Hyg. 56, 485–489. 10.4269/ajtmh.1997.56.485 9180594

[B61] Jabari S. da Silveira A. B. M. de Oliveira E. C. Neto S. G. Quint K. Neuhuber W.. (2011). Partial, selective survival of nitrergic neurons in chagasic megacolon. Histochem. Cell Biol. 135, 47–57. 10.1007/s00418-010-0774-y 21184236 PMC3019355

[B62] Jabari S. da Silveira A. B. M. de Oliveira E. C. Quint K. Neuhuber W. Brehmer A. (2012). Preponderance of inhibitory versus excitatory intramuscular nerve fibres in human chagasic megacolon. Int. J. Colorectal Dis. 27, 1181–1189. 10.1007/s00384-012-1500-0 22729712

[B63] Jabari S. da Silveira A. B. M. de Oliveira E. C. Quint K. Wirries A. Neuhuber W.. (2013). Interstitial cells of cajal: crucial for the development of megacolon in human chagas' disease? Colorectal Dis. 15, e592–e598. 10.1111/codi.12331 23810202

[B64] Jabari S. de Oliveira E. C. Brehmer A. da Silveira A. B. M. (2014). Chagasic megacolon: enteric neurons and related structures. Histochem. Cell Biol. 142, 235–244. 10.1007/s00418-014-1250-x 25059649 PMC4133073

[B65] Koberle F. (1968). Chagas' disease and chagas' syndromes: the pathology of American trypanosomiasis. Adv. Parasitol. 6, 63–116. 10.1016/S0065-308X(08)60472-8 4239747

[B66] Koberle F. de Alcantara F. (1960). Mechanism of destruction of the neurons of the peripheral nervous system in changas' disease. Hospital 57, 1057–1062. 14410284

[B67] Kulkarni G. V. Lee W. Seth A. McCulloch C. A. (1998). Role of mitochondrial membrane potential in concanavalin A-induced apoptosis in human fibroblasts. Exp. Cell Res. 245, 170–178. 10.1006/excr.1998.4245 9828113

[B68] Laranja F. S. Dias E. Nobrega G. Miranda A. (1956). Chagas' disease. Circulation 14, 1035–1060. 10.1161/01.CIR.14.6.1035 13383798

[B69] Leon J. S. Engman D. M. (2001). Autoimmunity in chagas heart disease. Int. J. Parasitol. 31, 555–561. 10.1016/S0020-7519(01)00163-1 11334942

[B70] Lopes G. P. Ferreira-Silva M. M. Ramos A. A. Moraes-Souza H. Prata A. Correia D. (2013). Length and caliber of the rectosigmoid colon among patients with chagas disease and controls from areas at different altitudes. Rev. Soc. Bras. Med. Trop. 46, 746–751. 10.1590/0037-8682-0247-2013 24474017

[B71] Lopez M. Tanowitz H. B. Garg N. J. (2018). Pathogenesis of chronic chagas disease: macrophages, mitochondria, and oxidative stress. Curr. Clin. Microbiol. Rep. 5, 45–54. 10.1007/s40588-018-0081-2 29868332 PMC5983038

[B72] Machado F. S. Dutra W. O. Esper L. Gollob K. J. Teixeira M. M. Factor S. M.. (2012). Current understanding of immunity to *Trypanosoma cruzi* infection and pathogenesis of chagas disease. Semin. Immunopathol. 34, 753–770. 10.1007/s00281-012-0351-7 23076807 PMC3498515

[B73] Maifrino L. Liberti E. de Souza R. (1999). Vasoactive-intestinal-peptide- and substance-P-immunoreactive nerve fibres in the myenteric plexus of mouse colon during the chronic phase of *Trypanosoma cruzi* infection. Ann. Trop. Med. Parasitol. 93, 49–56. 10.1080/00034989958799 10492671

[B74] Martins S. C. Lazarin-Bidóia D. Desoti V. C. Falzirolli H. da Silva C. C. Ueda-Nakamura T.. (2016). 1,3,4-thiadiazole derivatives of R-(+)-limonene benzaldehyde-thiosemicarbazones cause death in *Trypanosoma cruzi* through oxidative stress. Microbes Infect. 18, 787–797. 10.1016/j.micinf.2016.07.007 27484335

[B75] Massocatto C. L. Martins Moreira N. Muniz E. Marques de Araújo S. Pinge-Filho P. Rossi R. M.. (2017). Treatment with low doses of aspirin during chronic phase of experimental chagas' disease increases oesophageal nitrergic neuronal subpopulation in mice. Int. J. Exp. Pathol. 98, 356–362. 10.1111/iep.12259 29349896 PMC5826942

[B76] Meneghelli U. G. (1999). Clinical treatment of the digestive form of chagas disease. Mem. Inst. Oswaldo Cruz 94, 341–342. 10.1590/S0074-02761999000700066 10677752

[B77] Meneghelli U. G. (2004). Chagasic enteropathy. Rev. Soc. Bras. Med. Trop. 37, 252–260. 10.1590/S0037-86822004000300012 15330067

[B78] Meneghelli U. G. de Godoy R. A. Macedo J. F. de Oliveira R. B. Troncon L. E. Dantas R. O. (1982). Basal motility of dilated and non-dilated sigmoid colon and rectum in chagas' disease. Arq. Gastroenterol. 19, 127–132. 6821025

[B79] Meneghelli U. G. Godoy R. A. Oliveira R. B. Santos J. C. Dantas R. O. Troncon L. E. (1983). Effect of pentagastrin on the motor activity of the dilated and nondilated sigmoid and rectum in chagas' disease. Digestion 27, 152–158. 10.1159/000198945 6414867

[B80] Mirkin G. A. Celentano A. M. Malchiodi E. L. Jones M. González Cappa S. M. (1997). Different *Trypanosoma cruzi* strains promote neuromyopathic damage mediated by distinct T lymphocyte subsets. Clin. Exp. Immunol. 107, 328–334. 10.1111/j.1365-2249.1997.267-ce1166.x 9030871 PMC1904581

[B81] Mirkin G. A. Jones M. Sanz O. P. Rey R. Sica R. E. P. Cappa S. U. G. (1994). Experimental chagas' disease: electrophysiology and cell composition of the neuromyopathic inflammatory lesions in mice infected with a myotropic and a pantropic strain of *Trypanosoma cruzi*. Clin. Immunol. Immunopathol. 73, 69–79. 10.1006/clin.1994.1171 7923919

[B82] Moreira N. M. Sant'ana D. M. G. Araújo E. J. A. Toledo M. J. O. Gomes M. L. de Araújo S. M. (2011). Neuronal changes caused by *Trypanosoma cruzi*: an experimental model. An. Acad. Bras. Cienc. 83, 545–555. 10.1590/S0001-37652011000200014 21670878

[B83] Nascimento R. D. de Souza Lisboa A. Fujiwara R. T. de Freitas M. A. R. Adad S. J. Oliveira R. C.. (2010). Characterization of enteroglial cells and denervation process in chagasic patients with and without megaesophagus. Hum. Pathol. 41, 528–534. 10.1016/j.humpath.2009.05.018 20004942

[B84] Pérez-Molina J. A. Molina I. (2018). Chagas disease. Lancet 391, 82–94. 10.1016/S0140-6736(17)31612-4 28673423

[B85] Phillips R. J. Powley T. L. (2007). Innervation of the gastrointestinal tract: patterns of aging. Auton. Neurosci. 136, 1–19. 10.1016/j.autneu.2007.04.005 17537681 PMC2045700

[B86] Piacenza L. Alvarez M. N. Peluffo G. Radi R. (2009a). Fighting the oxidative assault: the *Trypanosoma cruzi* journey to infection. Curr. Opin. Microbiol. 12, 415–421. 10.1016/j.mib.2009.06.011 19616990

[B87] Piacenza L. Zago M. P. Peluffo G. Alvarez M. N. Basombrio M. A. Radi R. (2009b). Enzymes of the antioxidant network as novel determiners of *Trypanosoma cruzi* virulence. Int. J. Parasitol. 39, 1455–1464. 10.1016/j.ijpara.2009.05.010 19505468 PMC3909716

[B88] Pinto N. X. Torres-Hillera M. A. Mendoza E. León-Sarmiento F. E. (2002). Immune response, nitric oxide, autonomic dysfunction and stroke: a puzzling linkage on *Trypanosoma cruzi* infection. Med. Hypotheses 58, 374–377. 10.1054/mehy.2001.1401 12056871

[B89] Radi R. (2013). Peroxynitrite, a stealthy biological oxidant. J. Biol. Chem. 288, 26464–26472. 10.1074/jbc.R113.472936 23861390 PMC3772193

[B90] Radi R. (2018). Oxygen radicals, nitric oxide, and peroxynitrite: redox pathways in molecular medicine. Proc. Natl. Acad. Sci. U.S.A. 115, 5839–5848. 10.1073/pnas.1804932115 29802228 PMC6003358

[B91] Rassi A. Rassi A. Marcondes de Rezende J. (2012). American trypanosomiasis (chagas disease). Infect. Dis. Clin. North Am. 26, 275–291. 10.1016/j.idc.2012.03.002 22632639

[B92] Ribeiro U. Safatle-Ribeiro A. V. Habr-Gama A. Gama-Rodrigues J. J. Sohn J. Reynolds J. C. (1998). Effect of Chagas' disease on nitric oxide-containing neurons in severely affected and unaffected intestine. Dis. Colon Rectum 41, 1411–1417. 10.1007/BF02237058 9823808

[B93] Rivera L. R. Poole D. P. Thacker M. Furness J. B. (2011). The involvement of nitric oxide synthase neurons in enteric neuropathies. Neurogastroenterol. Motil. 23, 980–988. 10.1111/j.1365-2982.2011.01780.x 21895878

[B94] Rodrigues M. M. Alencar B. C. G. de Claser C. Tzelepis F. (2009). Immunodominance: a new hypothesis to explain parasite escape and host/parasite equilibrium leading to the chronic phase of chagas' disease? Braz. J. Med. Biol. Res. 42, 220–223. 10.1590/S0100-879X2009000300001 19287899

[B95] Santos S. L. Barcelos I. K. Mesquita M. A. (2000). Total and segmental colonic transit time in constipated patients with chagas' disease without megaesophagus or megacolon. Brazilian J. Med. Biol. Res. 33, 43–49. 10.1590/S0100-879X2000000100006 10625873

[B96] Shikanai-Yasuda M. A. Carvalho N. B. (2012). Oral transmission of chagas disease. Clin. Infect. Dis. 54, 845–852. 10.1093/cid/cir956 22238161

[B97] Smith T. H. Ngwainmbi J. Grider J. R. Dewey W. L. Akbarali H. I. (2013). An *in-vitro* preparation of isolated enteric neurons and glia from the myenteric plexus of the adult mouse. J. Vis. Exp. 7, e50688. 10.3791/50688 23962959 PMC3846983

[B98] Soares M. B. Pontes-De-Carvalho L. Ribeiro-Dos-Santos R. (2001). The pathogenesis of chagas' disease: when autoimmune and parasite-specific immune responses meet. An. Acad. Bras. Cienc. 73, 547–559. 10.1590/S0001-37652001000400008 11743602

[B99] Sun T. Li D. Hu S. Huang L. Sun H. Yang S.. (2018). Aging-dependent decrease in the numbers of enteric neurons, interstitial cells of cajal and expression of connexin43 in various regions of gastrointestinal tract. Aging 10, 3851–3865. 10.18632/aging.101677 30530917 PMC6326649

[B100] Tafuri W. L. Maria T. A. Lopes E. R. (1971). Myenteric plexus lesions in the esophagus, jejunum and colon of chronic chagasic patients. Electron microscopy study. Rev. Inst. Med. Trop. Saõ Paulo 13, 76–91. 5005737

[B101] Tanowitz H. B. Kirchhoff L. V. Simon D. Morris S. A. Weiss L. M. Wittner M. (1992). Chagas' disease. Clin. Microbiol. Rev. 5, 400–419. 10.1128/CMR.5.4.400 1423218 PMC358257

[B102] Tarleton R. L. (2001). Parasite persistence in the aetiology of chagas disease. Int. J. Parasitol. 31, 550–554. 10.1016/S0020-7519(01)00158-8 11334941

[B103] Teixeira A. R. L. Hecht M. M. Guimaro M. C. Sousa A. O. Nitz N. (2011). Pathogenesis of chagas' disease: parasite persistence and autoimmunity. Clin. Microbiol. Rev. 24, 592–630. 10.1128/CMR.00063-10 21734249 PMC3131057

[B104] Teixeira M. M. Gazzinelli R. T. Silva J. S. (2002). Chemokines, inflammation and *Trypanosoma cruzi* infection. Trends Parasitol. 18, 262–265. 10.1016/S1471-4922(02)02283-3 12036740

[B105] Vago A. R. Macedo A. M. Oliveira R. P. Andrade L. O. Chiari E. Galvão L. M.. (1996). Kinetoplast DNA signatures of *Trypanosoma cruzi* strains obtained directly from infected tissues. Am. J. Pathol. 149, 2153–2159. 8952547 PMC1865364

[B106] Vazquez B. P. Vazquez T. P. Miguel C. B. Rodrigues W. F. Mendes M. T. de Oliveira C. J. F.. (2015). Inflammatory responses and intestinal injury development during acute *Trypanosoma cruzi* infection are associated with the parasite load. Parasit. Vectors 8:206. 10.1186/s13071-015-0811-8 25889515 PMC4399205

[B107] Viader A. Wright-Jin E. C. Vohra B. P. S. Heuckeroth R. O. Milbrandt J. (2011). Differential regional and subtype-specific vulnerability of enteric neurons to mitochondrial dysfunction. PLoS ONE 6:e27727. 10.1371/journal.pone.0027727 22110743 PMC3218017

[B108] Vieira C. Godoy R. Meneghelli U. Carril C. (1996). Resposta do cólon sigmóide não ectásico à metacolina na forma crônica da moléstia de chagas. Arq. Gastroenterol. 16, 21–26.

[B109] Voukali E. Shotton H. R. Lincoln J. (2011). Selective responses of myenteric neurons to oxidative stress and diabetic stimuli. Neurogastroenterol. Motil. 23:964-e411. 10.1111/j.1365-2982.2011.01778.x 21914042

[B110] Wahba G. Hebert A.-E. Grynspan D. Staines W. Schock S. (2016). A rapid and efficient method for dissociated cultures of mouse myenteric neurons. J. Neurosci. Methods 261, 110–116. 10.1016/j.jneumeth.2015.11.024 26706461

[B111] Wang X. Michaelis E. K. (2010). Selective neuronal vulnerability to oxidative stress in the brain. Front. Aging Neurosci. 2:12. 10.3389/fnagi.2010.00012 20552050 PMC2874397

[B112] Wesley M. Moraes A. de Cássia Rosa A. Lott Carvalho J. Shiroma T. Vital T.. (2019). Correlation of parasite burden, kDNA integration, autoreactive antibodies, and cytokine pattern in the pathophysiology of chagas disease. Front. Microbiol. 10:1856. 10.3389/fmicb.2019.01856 31496999 PMC6712995

[B113] White R. J. Reynolds I. J. (1996). Mitochondrial depolarization in glutamate-stimulated neurons: an early signal specific to excitotoxin exposure. J. Neurosci. 16, 5688–5697. 8795624 10.1523/JNEUROSCI.16-18-05688.1996 PMC6578963

[B114] WHO (2018). Chagas Disease (American Trypanosomiasis). Tech. UNDP/World Bank/WHO. Available online at: http://www.who.int/news-room/fact-sheets/detail/chagas-disease-(american-trypanosomiasis) (accessed July 10, 2020).

[B115] Zhang L. Tarleton R. L. (1999). Parasite persistence correlates with disease severity and localization in chronic chagas' disease. J. Infect. Dis. 180, 480–486. 10.1086/314889 10395865

[B116] Zingales B. (2018). *Trypanosoma cruzi* genetic diversity: something new for something known about chagas disease manifestations, serodiagnosis and drug sensitivity. Acta Trop. 184, 38–52. 10.1016/j.actatropica.2017.09.017 28941731

