# Supplementary figures and images for: Neuronal Parasitism, Early Myenteric Neurons Depopulation and Continuous Axonal Networking Damage as Underlying Mechanisms of the Experimental Intestinal Chagas' Disease
Source: Front Cell Infect Microbiol. 2020 Oct 15;10:583899. doi: 10.3389/fcimb.2020.583899 (PMC7597600; doi:10.3389/fcimb.2020.583899)

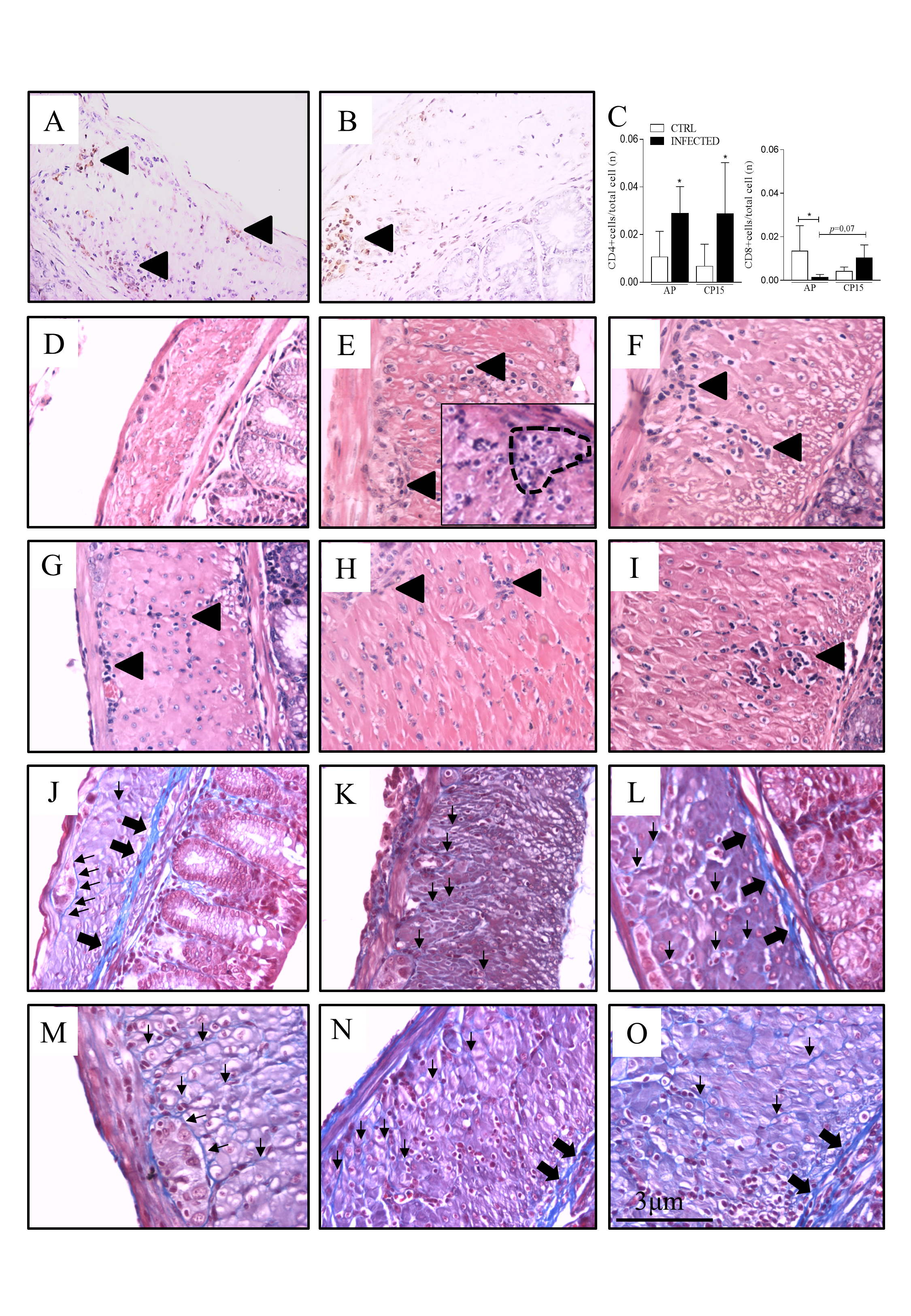

Supplement: Supplementary Figure 1 — Histopathological aspects of the colonic changes induced by the infection with the Y strain of T. cruzi. (A) CD4+ and (B) CD8+ cells observed in muscularis propria of chronic infected animals (15 m.p.i.) were quantified and expressed as (C) percentage of imunophenotyped (CD4+ and CD8+) cells/total cells of control and infected mice at acute (IAP, 11 d.p.i.) and chronic phase (ICP15, 15 m.p.i.). The percentage of CD4 + cells / total cells was higher for the infected groups when compared to the control groups (CTRL). The percentage of CD8 + cells / total cells decreased in IAP when compared to CTRL (CAP) animals, but increased in ICP15 mice (P = 0.07); (D–H) Histopathological aspects observed in colon samples of infected animals stained with HE where (D) CAP, (E) IAP, (F) ICP3, (G) ICP7, (H) ICP12, and (I) ICP15. Inflammatory foci were found scattered throughout the colon wall, at all times of the experiment. Notice that inflammatory cells in (E) (IAP) and (F) (ICP3) are associated with the vacuolization of parenchyma cells and hyper-eosinophilia. The infiltrate surrounds the enteric ganglia and sometimes blur its limits (inset, dashed line). Arrowhead indicates inflammatory infiltrate. (J) CTRL, (K) IAP, (L) ICP3, (M) ICP7, (N) ICP12, and (O) ICP15 Masson's trichome stains the connective tissue in blue. Reorganization and increased production of collagen fibbers throughout the infection. Thin arrows indicate interstitial and periganglionic collagen fibers, large arrows indicate collagen fibbers in the submucosal plexus. Scale Bar: 3 μm. 20X objective. Statistical analysis: ANOVA one-way with Student-Newman-Keuls post hoc tests. Difference in relation to the control group, P ≤ 0.05 (*) (n = 5 mice). Representative of two independent experiments. Data are shown as mean and and standard deviation (SD). [file Image_1.tif]
